# Phytochemistry and Biological Activities of Endophytic Fungi from the Meliaceae Family

**DOI:** 10.3390/molecules28020778

**Published:** 2023-01-12

**Authors:** Yeni Mulyani, Siska Elisahbet Sinaga, Unang Supratman

**Affiliations:** 1 Department of Chemistry, Faculty of Mathematic and Natural Sciences, Universitas Padjadjaran, Sumedang 45363, Indonesia; 2 Department of Marine Science, Faculty of Fisheries and Marine Science, Universitas Padjadjaran, Sumedang 45363, Indonesia; 3 Central Laboratory, Universitas Padjadjaran, Sumedang 45363, Indonesia

**Keywords:** endophytic fungi, Meliaceae, secondary metabolites, phytochemistry, bioactivities

## Abstract

Meliaceae plants are found worldwide in tropical or subtropical climates. They are important ethnobotanically as sources of traditional medicine, with 575 species and 51 genera. Previous research found that microorganisms are plant pioneers to produce secondary metabolites with diverse compound structures and bioactivities. Several plants of the Meliaceae family contain secondary metabolites isolated from endophytic fungi. Furthermore, related articles from 2002 to 2022 were collected from SciFinder, Google Scholar, and PubMed. About 276 compounds were isolated from endophytic fungi such as terpenoids, polyketides, lactones, pyrones, quinone, anthraquinones, xanthones, coumarines, isocoumarines, resorcylic acid lactones, cytochalasins, aromatics, ester, quinols, alkaloids, nitro compound, fatty acids, and sugars with bioactivities such as antioxidant, antibacterial, antifungal, anti-influenza, neuroprotective activities, anti-HIV, cytotoxic, allelopathic, anti-inflammatory, antifeedant effects, and BSLT toxicity. Meanwhile, secondary metabolites isolated from endophytic fungi were reported as one of the sources of active compounds for medicinal chemistry. This comprehensive review summarizes the ethnobotanical uses and secondary metabolites derived from Meliaceae endophytic fungi.

## 1. Introduction

Meliaceae is the mahogany family in tropical and subtropical regions such as the Himalayas, South America, Central America, Africa, South Asia, and Southeast Asia [1]. In this genus, the major secondary metabolites are sesquiterpenoid, triterpenoid, and limonoid, with minor compounds including flavonoid, lignans, chromone, and phenolic [2,3]. Many secondary metabolites that are valuable in pharmaceuticals have been isolated from the endophytic microorganisms of Meliaceae. Endophytes live in plant tissues without causing obvious disease at any time in the host. They produce bioactive substances that enhance the growth of the host plant. Endophytic fungi play important ecological roles in protecting plants from various biotic (pathogen damage) and abiotic stresses (such high salinity, temperature, and drought) [4,5,6].

Due to their extensive biological activities, fungal secondary metabolites possess unique chemical structures and are considered one of the best repositories for drug discovery from natural sources. Many endophytes produce bioactive products that block the growth of other organisms. In some cases, they can synthesize products similar to those produced by plants [7,8,9,10]. Plant growth promotion may be due to the ability to produce more growth-promoting metabolites [11,12]. Endophytic fungi are polyphyletic microorganisms that live in plant tissues, for examples *Fusarium fujikuroi*, *Colletotrichum* sp., and *Talaromyces verruculosus*. Generally, these microbial entities have been ignored as ecosystem components and are currently considered a new biodiversity richness. Endophyte biosynthetic study gained traction due to the discovery of capable strains synthesizing plant compounds [13,14,15]. Therefore, this study aims to provide an overview of the structural diversity of secondary metabolites in endophytic fungi. It focuses on the compounds found in Meliaceae endophytic fungi and their bioactivity. It also covers all compounds representing metabolic products isolated from endophytic fungi.

## 2. Methodology and Botany

This study started with a search for literature on the endophytic fungi isolated from the Meliaceae family and confirmed using a plant database (www.theplantlist.org (accessed on 29 November 2022)). Scifinder, PubMed, Google Scholar, Mendeley, and Scopus were used to collect articles on endophytic fungi isolated from Meliaceae based on biological and phytochemical properties. Meliaceae grows under lowland primary forests, particularly in Malesia, accounting for up to 17% of all trees with trunk diameters more significant than 10 cm found in Sumatran forests [16]. Some people actively seek out the bitterness of the bark, which has been known and used medicinally for centuries. One of these families includes the *Munronia pinnata* (Sapindales, Meliaceae) plant, a key ingredient in South Asian Materia Medica. The bark and leaves of *Azadirachta indica* (Rutales, Meliaceae), also known as neem, are effective insecticides, and the tree has a variety of applications, including the reclamation of abandoned land. The young shoots are used as vegetables (Sadao) and sold in markets worldwide, including Australia. Another example is *Lansium domesticum* (Sa-pindales, Meliaceae), which produces highly commercialized fruit used in traditional medicine as antidiarrheal, antimalarial, and anti-feeding drugs [17,18,19,20].

## 3. Phytochemistry

### 3.1. Overview of Isolated Compounds Derived from Endophytic Fungi Meliaceae

The Meliaceae family produces major secondary metabolites of the terpenoid group and minor secondary metabolites aromatic group. This can be seen in several plants belonging to the Meliaceae family, namely *Chisoseton macrophyllus*, which contains limonoids and triterpenoids; *Xylocarpus granatum* produces limonoids, protolimonoids, and flavonols; *Melia azedarach* shows flavonoids, terpenoids, steroids, and anthraquinones, and *Toona sinensis* contains terpenoids, phenylpropanoids, and flavonoids. *Azadirachta indica* produces alkaloids, steroids, flavonoids, and terpenoids; *Trichilia monedalpha* gives phenolic acids, terpenes, steroids, and limonoids. At the same time, *Dysoxylum* and *Lansium domesticum* produce triterpenoids, and limonoid, sesquiterpenoid, and steroid compounds. On the other hand, *Swietenia* contains terpenoid compounds, the most dominant of which are lignans and limonoids.

**Figure 1:**
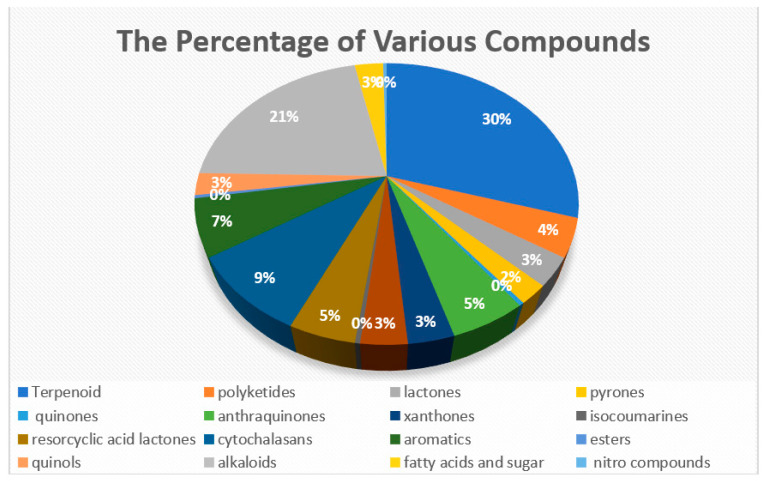
The percentage of various compounds which have been isolated from endophytic fungi in Meliaceae [31,32,33,34,35,36,37,38,39,40,41,42,43,44,45,46,47,48,49,50,51,52,53,54,55,56,57,58,59,60,61,62,63].

The group of compounds successfully isolated from the Meliaceae family showed a relationship with the group of compounds successfully isolated from endophytic fungi. This is shown by the compounds that were successfully isolated, and all of them entered into a group that had been successfully isolated from their host. The 276 compounds have been isolated from the endophytic fungi from the stembark, leaves, fruits, barks, seeds, and roots of Meliaceae, based on the literature from 2002 to 2022 (Table S1). *C. macrophyllus* [21], *X. granatum* [22], *M. azedarach* [23], *T. sinensis* [24], *C. tagal* [25], *A. indica* [26], *T. monadelpha* [27], *T. longipes* [28], *D. binectariferum* [29], and *L. domesticum* [30] are members of the Meliaceae family with endophytic fungi that create secondary metabolites. The 276 secondary metabolites isolated are composed of 82 terpenoids, 12 polyketides, nine lactones, six pyrones, one quinone, 15 anthraquinones, nine xanthones, 10 isocoumarines, 13 resorcylic acid lactones, 25 cytochalasins, 18 aromatics, one ester, seven quinols, 59 alkaloids, one nitro compound, and eight fatty acids and sugars (Figure 1) [31,32,33,34,35,36,37,38,39,40,41,42,43,44,45,46,47,48,49,50,51,52,53,54,55,56,57,58,59,60,61,62,63,64].

### 3.2. Triterpenoid and Sesquiterpenoid Compounds

**Figure 2:**
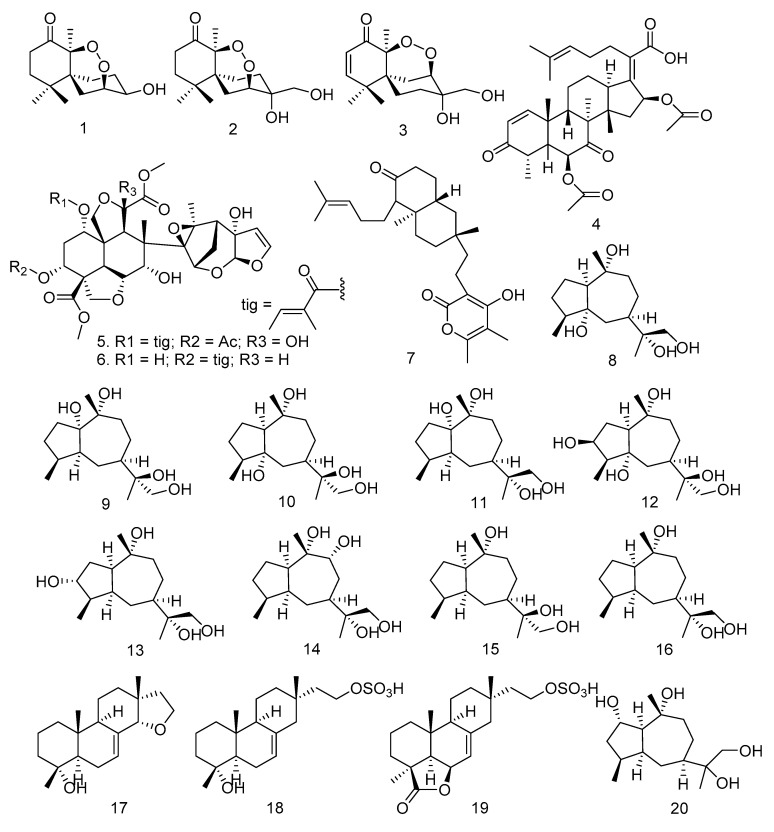

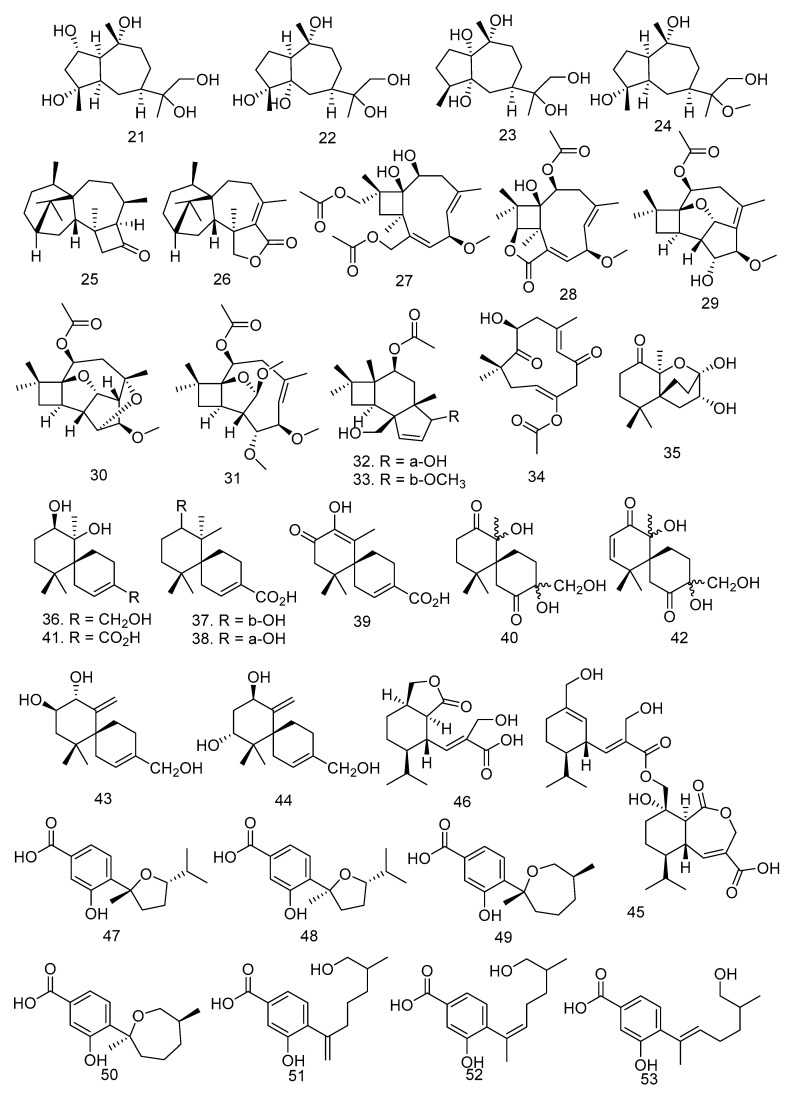

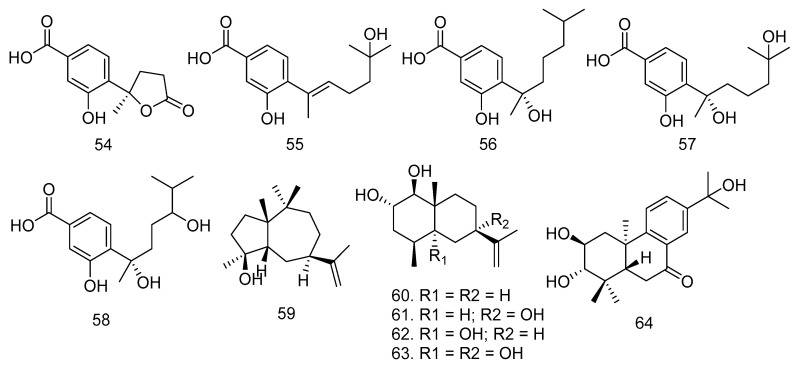
Triterpenoids, diterpenoids, and sesquiterpenoids were obtained from endophytic fungi isolated from the Meliaceae family.

Based on previous study, 82 terpenoids have been isolated from endophytic fungi isolated from the Meliaceae family. The isolated terpenoids are classified into sesquiterpenoids, triterpenoids, steroids, and meroterpenoids. A type of sesquiterpenoid obtained is the Chamigrane reported by Chookpaibon et al. (2010) [31]. Furthermore, three Chamigrane endoperoxides (Figure 2) were isolated from the XG8D fungus, which was taken from leaves of *X. granatum* and fermented in steep corn liquor containing medium under static conditions. These compounds are Merulin A (**1**), Merulin B (**2**), and Merulin C (**3**). They were also isolated from several species of marine algae of the genus *Laurencia* [64,65,66,67,68,69,70]. 

Li et al. [32] isolated the phytotoxic nordammarane triterpenoid helvolic acid (**4**) from the *Aspergillus fumigatus* LN-4 fungus of stembark *M. azedarach*. Azadirachtin A (**5**) and B (**6**) were obtained from the *E. parvum* fungus, taken from seeds, leaves, stem/twigs, inner bark, and roots of *A. indica* A. J_USS_ [33]. On the other hand, Xiao et al. [34] successfully isolated Pycnophorin (**7**) from the *B. dothidea* KJ-1 strain cultured on PDA and produced from the chloroform fraction. The endophytic fungal strain KJ-1 was obtained from the symptomless tissue of the stem bark of *M. azedarach* L. [34]. An endophytic fungus, *Xylaria* sp. YM 311647, isolated from *A. indica*, has produced nine new oxygenated guaiane-type sesquiterpenes (**8**–**16**) and three new isopimarane diterpenes (**17**–**19**). These compounds are (1*S*,4*S*,5*R*,7*R*,10*R*,11*R*)-guaiane-5,10,11,12-tetraol (**8**), (1*S*,4*S*,5*S*,7*R*,10*R*,11*S*)-guaiane-1,10,11,12-tetraol (**9**), (1*S*,4*S*,5*R*,7*R*,10*R*,11*S*)-guaiane-5,10,11,12-tetraol (**10**), (1*S*,4*S*,5*S*,7*R*,10*R*,11*R*)-guaiane-1,10,11,12-tetraol (**11**), (1*R*,3*S*,4*R*,5*S*,7*R*,10*R*,11*S*)-guaiane-3,10,11,12-tetraol (**12**), (1*R*,3*R*,4*R*,5*S*,7*R*,10*R*,11*R*)-guaiane-3,10,11,12-tetraol (**13**), (1*R*,4*S*,5*S*,7*S*,9*R*,10*S*,11*R*)-guaiane-9,10,11,12-tetraol (**14**), (1*R*,4*S*,5*S*,7*R*,10*R*,11*S*)-guaiane-10,11,12-triol (**15**), (1*R*,4*S*,5*S*,7*R*,10*R*,11*R*)-guaiane-10,11,12-triol (**16**), 14α,16-epoxy-18-norisopimar-7-en-4α-ol (**17**), 16-*O*-sulfo-18-norisopimar-7-en-4α,16-diol (**18**), and 9-deoxy-hymatoxin A (**19**) [35]. Furthermore, Figure 2 shows the diagram of compounds **1**–**19**. 

Li et al. [37] succeeded in isolating compounds from *A. indica*. Huang also successfully obtained five guaian-type sesquiterpenes from the fungus *Xylaria* sp. YM 311647. The five compounds are guaiane-2,10,11,12-tetraol (**20**), guaiane-2,4,10,11,12-pentaol (**21**), guaiane-4,5,10,11,12-pentaol (**22**), guaiane-1,5,10,11,12-pentaol (**23**), and 11-methoxyguaiane-4,10,12-triol (**24**) [36]. The leaves, stems, and bark of the mangrove plant *Xylocarpus granatum* produced the fungus *Trichoderma* sp. Xy24. It was fermented in PDA liquid media to isolate two new compounds of the diterpenoid type, namely (9*R*, 10*R*)-dihydroharzianone (**25**) and Harzianelactone (**26**) [37]. 

Caryophyllene sesquiterpenoids such as pestaloporinates A-G (**27**–**33**) and 14-acetylhumulane (**34**) were isolated from *Pestalotiopsis* sp. by an 18S rDNA sequence, which was obtained from the fresh stem bark of *M. azedarach* Linn and cultured in PDA [38]. In addition, six new chamigrane sesquiterpenes, merulinols A-F (**35**–**40**) and known compounds, aciicolinol C (**41**), aciicolinol K (**42**), aciicolinol F (**43**), and aciicolinol D (**44**), were obtained from the culture of an endophytic fungus XG8D isolated from the fresh leaves of a *X. granatum* plant in Thailand [39].

Colletotrin (**45**), a new sesquiterpene lactone, and one known compound, hydroheptelidic acid (**46**), were isolated from a rice culture of *C. gloeosporioides*, an endophytic fungus obtained from the stem bark of the Cameroonian medicinal plant *T. monadelpha* (Meliaceae) [27]. Meanwhile, seven new phenolic bisabolane sesquiterpenoids such as (7*R*,10*S*)-7,10-epoxysydonic (**47**), (7*S*,10*S*)-7,10-epoxysydonic (**48**), (7*R*,11*S*)-7,12-epoxysydonic (**49**), (7*S*,11*S*)-7,12-epoxysydonic (**50**), 7-deoxy-7,14-didehydro-12-hydroxysydonic (**51**), and (*Z*)-7-deoxy-7,8-didehydro-12-hydroxysydonic acid (**52**), and six known compounds, (*E*)-7-deoxy-7,8-didehydro-12-hydroxysydonic (**53**), (+)-1-hydroxyboivinianic (**54**), engyodontiumone I (**55**), (+)-sydonic (**56**), (+)-hydroxysydonic (**57**), (−)-(7*S*)-10-hydroxysydonic acid (**58**), were obtained from the culture of an endophytic fungus *Aspergillus* sp. xy02 isolated from the leaves of a Thai mangrove, *Xylocarpus moluccensis* [40]. 

The other sesquiterpenoid isolated from *Xylaria* sp. HNWSW-2, the stem of *X. granatum*, is guaidiol (**59**) [41]. In 2018, Qiu et al. [25] isolated four new eudesmane-type sesquiterpenoids, penicieudesmol A-D (**60**–**63**), from the fermentation broth of the endophytic fungus *Penicillium* sp. J-54 derived from mangroves. *Penicillium* sp. J-54 was isolated from the healthy leaves of the *Ceriops tagal*, which were collected in the Dong Zhai Gang Mangrove [25]. Meanwhile, a bietanetype-diterpenoid, hydroxyldecandrin G (**64**), was isolated from *Xylaria* sp. XC-16, which is obtained from leaves of *T. sinensis*. The structures of compounds **20**–**64** can be seen in Figure 2.

### 3.3. Steroid Compounds

Ergokonin B (**65**) and cerevisterol (**66**) were isolated from an endophytic fungus, *Fusarium* sp. LN-11, from the leaves of *M. azedarach* [21]. Cerevisterol was also obtained from *F. phaseoli* [43]. In the study by Xiao et al. [34], *B. dothidea* KJ-1 was isolated from the stem of *M. azedarach*, and Sari et al. [43] stated that *F. phaseoli* from the root of *C. macrophyllus* produced ergosterol peroxide (**67**). β-sitosterol glucoside (**68**) was isolated from *B. dothidea* KJ-1 found in the stem of *M. azedarach* L. [34]. Ergosterol (**69**) was obtained from *Eupenicillium* sp. HJ002 derived from *X. granatum* and *F. phaseoli* derived from *C. macrophyllus* [22,43].

**Figure 3:**
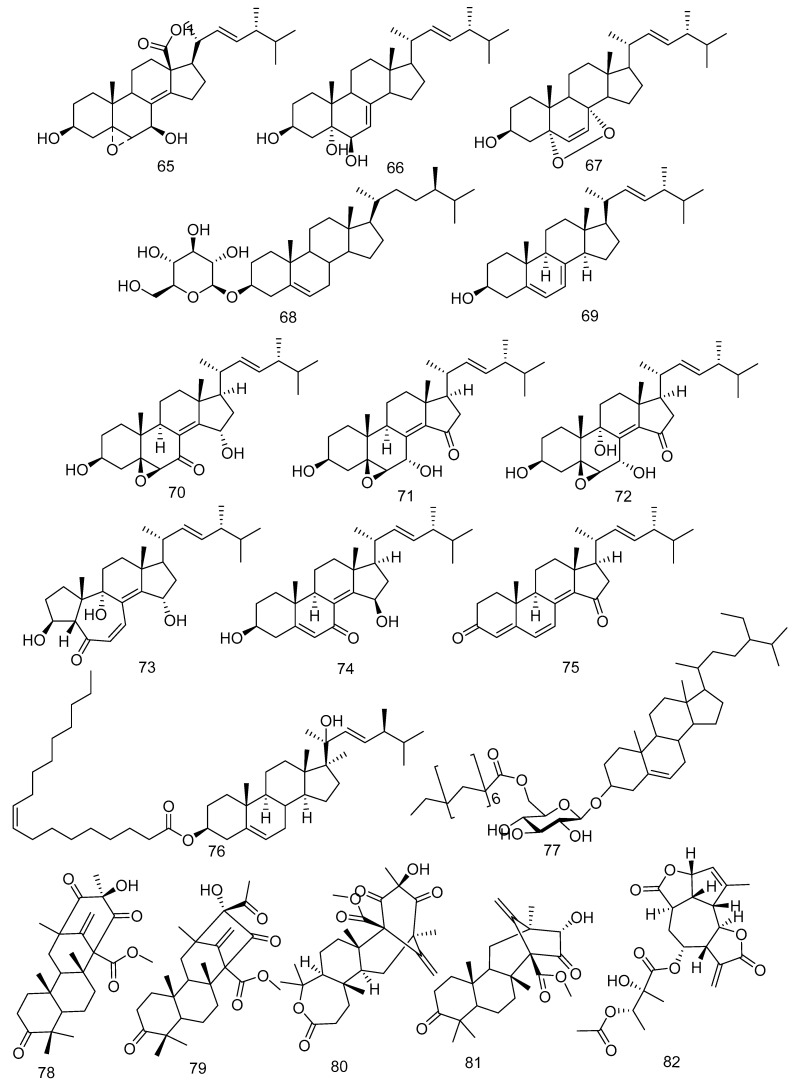
The steroid (**65**–**77**) and meroterpenoid (**78**–**82**) compounds were obtained from various fungi in Meliaceae Family.

A new αβ-unsaturated 7-ketone sterol, 5β,6β-epoxy-3β,15α-dihydroxy-(22*E*,24*R*)-ergosta-8(14),22-dien-7-one (**70**), with five known sterone derivatives, 5β,6β-epoxy-3β,7α-dihydroxy(22*E*,24*R*)-ergosta-8(14),22-dien-15-one (**71**), 5β,6β-epoxy-3β,7α,9a-trihydroxy-(22*E*,24*R*)-ergosta-8(14),22-dien-15-one (**72**), 3β,9α,15a-trihydroxy-(22*E*,24*R*)-10(5→4)abeo-ergosta-6,8(14),22-trien-5-one (**73**), 3,15-dihydroxyl-(22*E*,24*R*)-ergosta-5,8(14),22trien-7-one (**74**), (22*E*,24*R*)-ergosta-4,6,8(14),22-tetraen-3,15-dione (**75**), were isolated from the mangrove-derived fungus *Phomopsis* sp. MGF222 [45]. In addition, a new ergostane-type sterol, ergost-5,22*E*-dien-3-oleate-20-ol (**76**), and one known atroside (**77**) were obtained from the solid brown rice culture of *F. phaseoli*, an endophytic fungus found in the root of *C. macrophyllus* [43]. The structures of the steroid compounds **65**–**77** can be seen in Figure 3.

### 3.4. Meroterpenoid Compounds

In the study conducted by Geris dos Santos and Rodrigues-Fo. [46], a *Penicillium* sp. isolated from the root bark of *M. azedarach* and cultivated over sterilized rice produced two meroterpenes, namely preaustinoid A (**78**) and B (**79**). In addition to a previous study about meroterpenoids, Fill et al. [47] reported that three known meroterpenoids, preaustinoid A1 (**80**), preaustinoid B2 (**81**), and austinolide (**82**), have been isolated from *Penicillium brasilianum* found in the root bark of *Melia azedarach* (Figure 3).

### 3.5. Polyketides

Aurasperone A (**83**) and fonsecinone A (**84**), the polyketides, were isolated from *Aspergillus aculeatus*, an endophytic fungus in the leaves of *M. azedarach* [48]. Dianhydro-aurasperone C (**85**), isoaurasperone A (**86**), fonsecinone A (**87**), asperpyrone A (**88**), and rubrofusarin B (**89**) were obtained from *Aspergillus* sp. KJ-9 fungi isolated from stembark of *M. azedarach.* In addition to **83**–**89**, the *Botryosphaeria dothidea* KJ-1 found in the stem of this family also produced Stemphyperylenol (**90**) [34,44]. 

**Figure 4:**
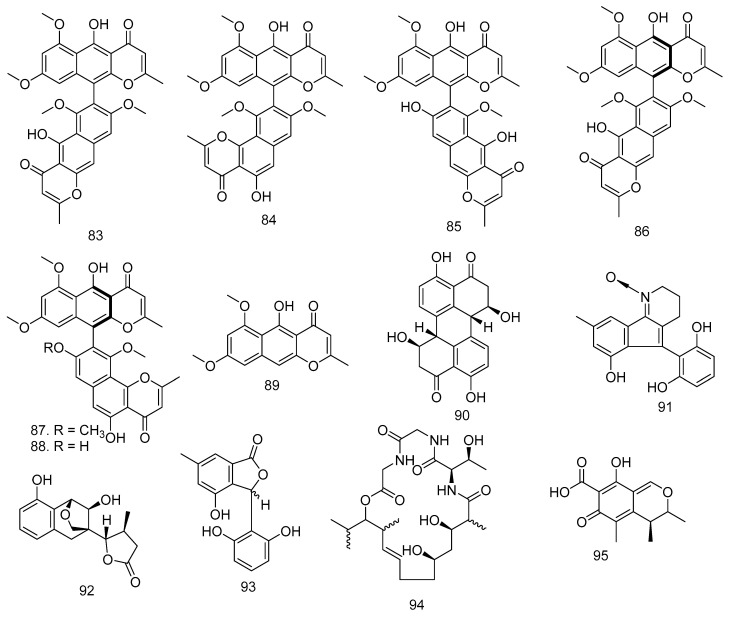
The compounds of polyketides obtained from various fungi in the Meliaceae Family.

The Thai mangrove endophytic fungus *Phomopsis* sp. xy21 produced phomopsol A (**91**), a polyketide-derived alkaloid with a unique 3,4-dihydro-2*H*-indeno [1,2-b]pyridine 1-oxide motif, phomopsol B (**92**), which is a highly oxidized polyketide with a new 3,5-dihydro-2*H*-2,5-methanobenzo[e][1,4]-dioxepine moiety, and 3-(2,6-dihydroxyphenyl)-4-hydroxy- 6-methylisobenzofuran-1(3*H*)-one (**93**) [49]. Gao et al. [50] reported that Eucalactam B (**94**) was obtained from *D. eucalyptorum* KY-9 fungi found in the leaves of *M. azedarach* [50]. Citrinin (**95**) is a well-known mycotoxin produced mainly by *Penicillium citrinum* and several *Aspergillus* species. This polyketide was obtained in good yields by *P. janthinellum* from the host plant *M. azedarach* [51,71], and the structure can be found in Figure 4.

### 3.6. Lactones

**Figure 5:**
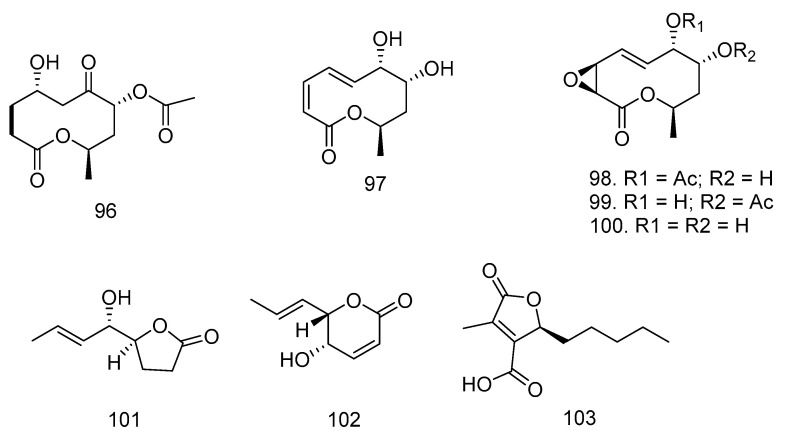
Lactone structures isolated from Meliaceae-derived fungi.

In addition to producing the azadiractin type, Wu et al. [52] reported in 2008 that the plant *A. indica* also produces four new lactones (8α-acetoxy-5α-hydroxy-7-oxodecan-10-olide (**96**), 7α,8α-dihydroxy-3,5-decadien-10-olide (**97**), 7α-acetoxymultiplolide A (**98**), and 8α-acetoxymultiplolide A (**99**). A known lactone, multiplolide A (**100**) was isolated from broth extracts of the endophytic mushroom *Phomopsis* sp. from the stem of *A. indica*. Meanwhile, in 2009, Wu et al. [52] obtained nigrosporalactone (**101**) and phomalactone (**102**) from the fermentation culture of *Nigrospora* sp. YB-141, an endophytic fungus isolated from *A. indica*. In addition to Wu, Mei et al. [22] successfully isolated a lactone compound, (*R*)-striatisporolide A (**103**), from the *X. granatum* Koenig-derived fungus *Eupenicillium* sp. HJ002 for the first time. Lactone structures can be found in Figure 5.

### 3.7. Pyrones, Quinone, and Anthraquinones

**Figure 6:**
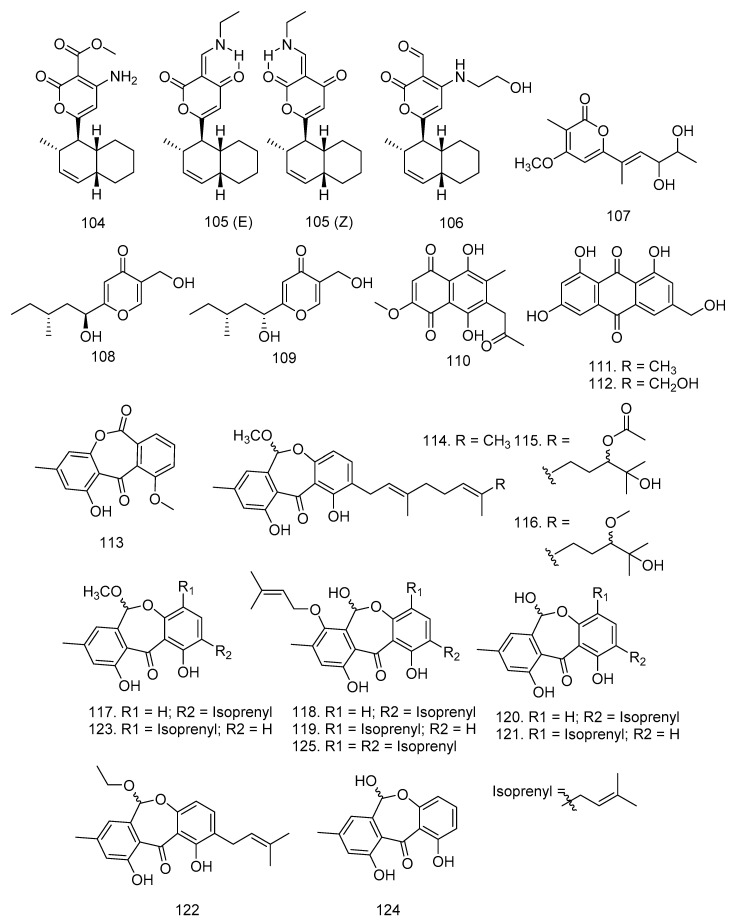
Pyrones (**104**–**109**), quinone (**110**), and anthraquinones (**111**–**125**) were isolated from Meliaceae-derived fungi.

In addition to lactones, Wu et al. [52] also succeeded in proving that *Nigrospora* sp. YB-141, an endophytic fungus isolated from *A. indica*, produced two new pyrone compounds, solanapyrone N (**104**) and solanapyrone O (**105**), and one known compound, solanapyrone C (**106**). Meanwhile, Wang et al. [41] obtained astropyrone (**107**), xylaropyrone B (**108**), and xylaropyrone C (**109**) from the fermentation broth of *Xylaria* sp. HNWSW-2 fungus in *X. granatum* stem (Figure 6).

Endophytic fungi also produced quinone and anthraquinones. Kharwar et al. [53] isolated the quinone type, namely javanicin (**110**), from *Chloridium* sp. fungi found in *A. indica* root [53]. New anthraquinones such as emodin (1,6,8-trihydroxy-3-methylanthra-quinone) (**111**), citreorosein (1,6,8-trihydroxy-3-methylanthraquinone) (**112**), and janthinone (**113**) were obtained from *P. janthinellum*, isolated as an endophytic fungus from *M. azedarach* fruits grown on the ground for 20 days and autoclaved white corn [51,54].

Endophytic fungi Xylariaceae were isolated from fresh and healthy leaves of *L. domesticum* collected on tropical peatland in West Kalimantan using ITS sequencing. Internal transcribed spacer (ITS) is a piece of nonfunctional RNA located between structural ribosomal RNAs (rRNA) of a common precursor transcript, which is especially useful for elucidating relationships among congeneric species and closely related genera. Chromatographic separation of the ethyl acetate extract yielded three new arugosin-type metabolites, including arugosins O (**114**), P (**115**), and Q (**116**), as well as nine known compounds such as arugosin K (**117**), arugosin A (**118**), arugosin B (**119**), arugosin N (**120**), 1,6,10-trihydroxy-8-methyl-2-(3-methyl-2-butenyl)-dibenz[b,e]oxepin-11(6*H*)-one (**121**), arugosin L (**122**), arugosin M (**123**), arugosin F (**124**), and arugosin G (**125**) [54]. The structures of quinone and anthraquinone compounds are shown in Figure 6.

### 3.8. Xanthones, Isocoumarin, and Resorcylic Acids

**Figure 7:**
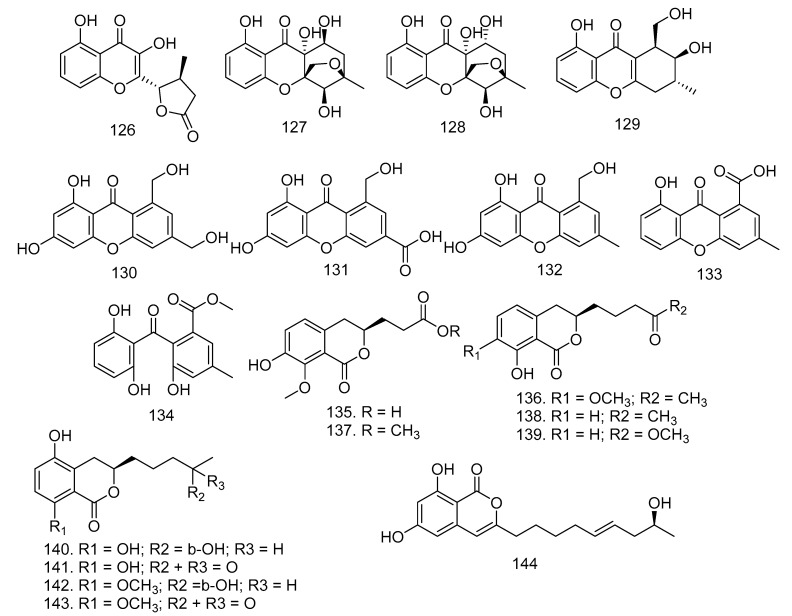
Xanthones (**126**–**134**) and isocoumarins (**135**–**144**), isolated from Meliaceae-derived fungi.

Based on the endophytic research fungi conducted by Hu et al. [55] six new xanthone-derived polyketides, phomoxanthones F-K (**126**–**131**), as well as three known xanthones, leptosphaerin E (**132**), mono-dictyxanthone(8-hydroxy-3-methyl-9-oxo-9*H*-xanthene-1-carboxylic acid) (**133**), and 2,2′,6′-trihydroxy-4-methyl-6-methoxy-acyl-diphenylmethanone (**134**), were isolated from *Phomopsis* sp. xy21, an endophytic fungus in the Thai mangrove *X. granatum* (Figure 7).

In addition to xanthones, two new isocoumarins, penicimarins L-N (**135**–**136**), and seven known isocoumarins, peniisocoumarin E (**137**), apergilumarin A (**138**), penicimarin I (**139**), peniisocoumarin F (**140**), penicilloxalone B (**141**), penicimarin G (**142**), and penicimarin H (**143**), were successfully obtained from the endophytic fungus *Penicillium* sp. MGP11 in *X. granatum* [56]. Fusariumin (**144**) was isolated from *Fusarium* sp. LN-10 in the leaves of *M. azedarach* (Figure 7) [57].

**Figure 8:**
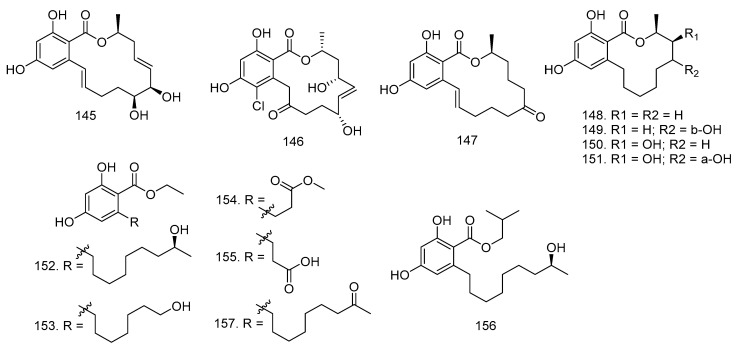
Compounds of resorcylic acid derivatives isolated from Endophytic fungi in Meliaceae.

In addition to fusariumin (**144**), Yang et al. [57] also isolated two known resorcylic acid lactones, aigialomycin D (**145**), pochonin N (**146**), and zearalenone (**147**), from the cultures of *Fusarium* sp. LN-10. The study conducted by Sato et al. [58] reported that the endophytic fungus *L. theobromae* in the mangrove plant *X. granatum* produced nine new -resorcylic acid derivatives such as (15*S*)-de-*O*-methyllasiodiplodin (**148**), (13*S*,15*S*)-13-hydroxy-de-*O*-methyllasiodiplodin (**149**), (14*S*,15*S*)-14-hydroxy-de-*O*-methyllasiodiplodin (**150**), (13*R*,14*S*,15*S*)-13,14-dihydroxy-de-*O*-methyllasiodiplodin (**151**), ethyl (*S*)-2,4-dihydroxy-6-(8-hydroxynonyl)benzoate (**152**), ethyl 2,4-dihydroxy-6-(8-hydroxyheptyl) benzoate (**153**), ethyl 2,4-dihydroxy-6-(4-methoxycarbonylbutyl)benzoate (**154**), 3-(2-ethoxycarbonyl-3,5-dihydroxyphen-yl)propionic acid (**155**), (*S*)-2,4-dihydroxy-6-(8-hydroxynonyl)benzoate (**156**), and one known compound, ethyl 2,4-dihydroxy-6-(8-oxononyl)benzoate (**157**) (Figure 8).

### 3.9. Cytochalasins

In the study by Zhang et al. [34], the fermentation extract of *Xylaria* sp. XC-16 from *T. sinensis* produced two new cytochalasins, cytochalasin Z_27_ (**158**) and cytochalasin Z_28_ (**159**), and three known compounds seco cytochalasin E (**160**), cytochalasin Z_18_ (**161**), and cytochalasin E (**162**) [24]. Chaetoglobosins C (**163**) and F (**164**) were isolated from a solid culture of the endophytic fungus *B. dothidea* KJ-1, collected from white cedar stems (*M. azedarach* L.).

*Phomopsis* spp. xy21 and xy22, from Thai mangrove fungal endophytes, produced four new cytochalasins, phomopsichalasins D-G (**165**–**168**), and six known ones, namely 4′-hydroxy-deacetyl-18-deoxycytochalasin H (**169**), deacetyl-18-deoxycytochalasin H (**170**), 18-deoxycytochalasin H (**171**), cytochalasin H (**172**), deacetylcytochalasin H (**173**), and epoxycytochalasin H (**174**) [59,72,73,74]. Cytochalasin D (**175**) was obtained from *C. gloeosporioides* in the stem bark of *T. monadelpha* [27]. Xylarisin B (**176**) was produced by *Xylaria* sp. HNWSW-2, isolated from the stem of *X. granatum* [41].

**Figure 9:**
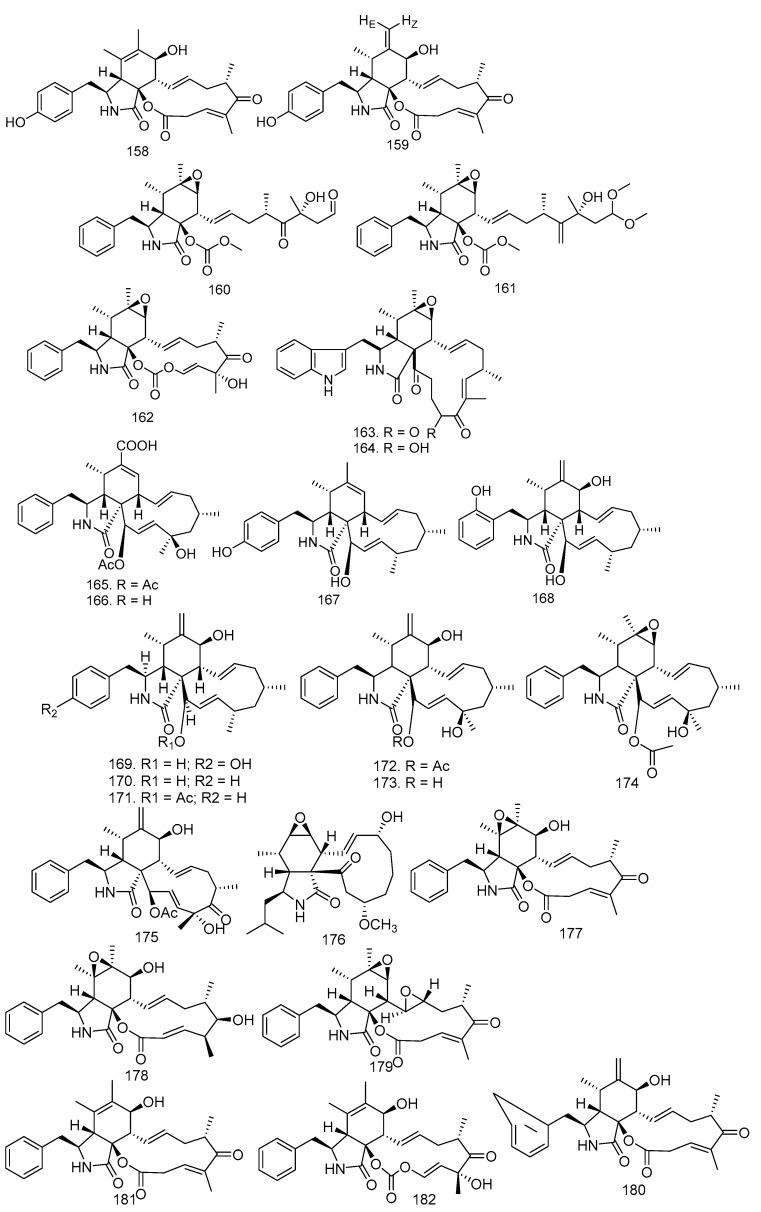
All of the cytochalasin structures obtained from endophytic fungi in Meliaceae.

In 2019, Han et al. [42] conducted research on *Xylaria* sp. XC-16 isolated from *T. sinensis* leaves to yield epoxycytochalasin Z_17_ (**177**), epoxycytochalasin Z_8_ (**178**), epoxyrosellichalasin (**179**), 10-phenyl-[12]-cytochalasin Z_16_ (**180**), 10-phenyl-[12]-cytochalasin Z1_7_ (**181**), and cytochalasin K (**182**). All of the cytochalasin structures can be seen in Figure 9.

### 3.10. Aromatics, Ester, Quinols

**Figure 10:**
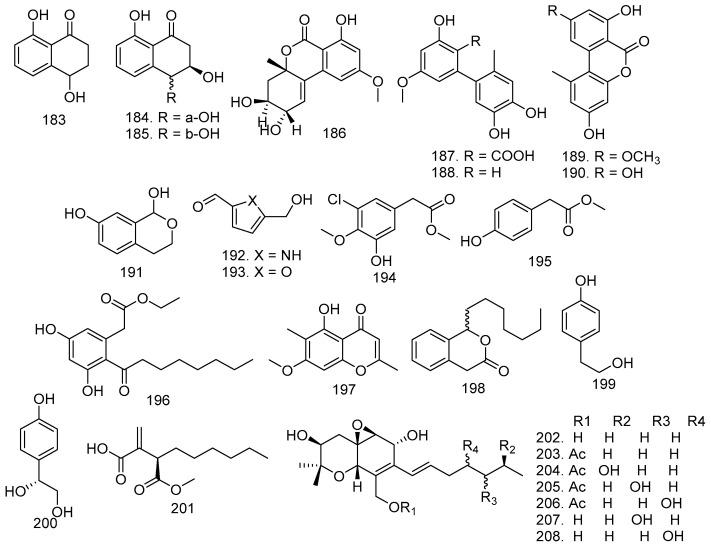
All structures of aromatics (**183**–**200**), ester (**201**), and quinols (**202**–**208**) produced by endophytic fungi from the Meliaceae family.

The stem bark of *M. azedarach* has *A. fumigatus* LN-4, cultured in PD liquid medium. The fungus produced 4,8-dihydroxy-1-tetralone (**183**), *trans*-3,4-dihydro-3,4,8-trihydroxynaphtalen-1(2*H*)-one (**184**), and *cis*-3,4-dihydro-3,4,8-trihydroxynaphtalen-1(2*H*)-one (**185**) [32]. In addition, this family also has the *B. dothidea* KJ-1 fungus, which produced altenuene (**186**), (**187**), djalonensone (**188**), alternariol (**189**), 5-methoxy-6-methylbiphenyl-3,4,3-triol (**190**), 7-hydroxy-1-isochromanone3587 (**191**), 5-(hydroxymethyl)-1*H*-pyrrole-2-carbaldehyde (**192**), and 5-hydroxymethylfurfural (**193**) (Figure 10) [34].

The fungal strain *Eupenicillium* sp. HJ002 was obtained from the mangrove *Xylocarpus granatum* Koenig in the South China Sea. Extracts of this fungus yielded new aromatic compounds 3-chloro-5-hydroxy-4-methoxyphenylacetic acid methyl ester (**194**) and two known derivatives, 4-hydroxyphenylacetate (**195**) and cytosporone B (**194**) [22]. Apart from Xiao et al. [44], Gao et al. [50] also observed that *Eucalyptorum* KY-9 was isolated from the leaves of *M. azaderach* plant and cultured in rice. The endophytic fungus yielded eugenitol (**197**), cytosporone C (**198**), 4-hydroxyphenethyl alcohol (**199**), and 1-(4-hydroxyphenyl)ethane-1,2-diol (**200**) (Figure 10).

(*R*)-butanedioic acid (**201**) ester was produced by *Eupenicillium* sp. HJ002, which was isolated from *X. granatum* [22,50]. The leaves of *T. longipes* delivered *P. theae* fungi, which gave six new epoxyquinols, cytosporins F-K (**203**–**208**), with the known cytosporin D (**202**) (Figure 10) [75].

### 3.11. Alkaloids

*Penicillium* sp. was isolated from the root bark of *M. azedarach* and grown on sterilized rice. The known alkaloid verruculogen (**209**) was obtained after chromatographic procedures. Apart from *Penicillium* sp., *A. fumigatus* LN-4 obtained from *M. azedarach* stem bark and cultured in PD liquid medium also yielded this compound [32,46]. In the study conducted by Li et al. [49], brasiliamide A (**210**) and brasiliamide B (**211**) were produced from *P. brasilianum* in the root bark of *M. azaderach* [47,60].

Alkaloids **212**–**245** were isolated from the fermentation broth of *Aspergillus fumigatus* LN-4, an endophytic fungus obtained from the stem bark of *Melia azedarach*, including 12β-hydroxy-13α-methoxyverruculogen TR-2 (**217**), 3-hydroxyfumiquinazoline A (**226**) and 32 known alkaloids such as fumitremorgin C (**212**), cyclotryprostatin A (**213**), cyclotryprostatin B (**214**), verrulocogen TR-2 (**215**), 12β-hydroxyverruculogen TR-2 (**216**), 12β-hydroxy-13α-methoxyverruculogen TR-2 (**217**), fumitremorgin B (**218**), tryprostatin A (**219**), cyclo-l-tryptophyl-l-proline (**220**), terezine D (**221**), fumiquinazoline F, G, D, A (**222**–**225**), 3-hydroxyfumiquinazoline A (**226**), 6-methoxyspirotryprostatin B (**227**), spiro [5*H*,10*H*-dipyrrolo [1,2-α:1′,2′-d]pyrazine-2-(3*H*),2′-[2*H*]indole]-3′,5,10(1′*H*)-trione (**228**), pseurotin A (**229**), pseurotin A1 (**230**), tryptoquivaline O (**231**), fumifaclavine B (**232**), bisdethiobis(methylthio)gliotoxin (**233**), cyclo-(Pro-Gly) (**234**), cyclo-(Pro-Ala) (**235**), cyclo-(d-Pro-l-Ala) (**236**), cyclo-(Pro-Ser) (**237**), cyclo-(Ser-*trans*-4-OH-Pro) (**238**), cyclo-(Leu-4-OH-Pro) (**239**), cyclo-(Ala-*trans*-4-OH-Pro) (**240**), cyclo-(*cis*-OH-d-Pro-l-Phe) (**241**), cyclo-(Gly-Phe) (**242**), cyclo-(Pro-tans-4-OH-Pro) (**243**), cyclo-(Gly-Ala) (**244**), and uracil (**245**) [32].

Further study was conducted by Kumara et al. [61] where rohitukine (**246**) was obtained from several endophytic fungi isolated from *D. binectariferum* Hook. f, such as *F. proliferatum* MTCC 9690, *F. oxysporum* MTCC 11383*, F. solani* MTCC 11385, *F. oxysporum* MTCC 11384, and *G. fujikurai* MTCC 11382 from the bark, leaves, fruit, and bark respectively. Apart from compound **246**, rohitukine *N*-oxide (**247**) and flavopiridol (**248**) were also isolated from these endophytic fungi. 

In 2014, Wang [62] also discovered 13 alkaloids, one of which was a new alkaloid 2-(furan-2-yl)-6-(2*S*,3*S*,4-trihydroxybutyl)pyrazine (**249**) and 12 known compounds such as 2-(furan-2-yl)-5-(2*S*,3*S*,4-trihydroxybutyl)pyrazine (**250**), (*S*)-4-isobutyl-3-oxo-3,4-dihydro-1-*H*,pyrrolo [2,1-c][1,4]oxazine-6-carbaldehyde (**251**), (*S*)-4-isopropyl-3-oxo-3,4-dihydro-1*H*-pyrrolo [2,1-c][1,4]oxazine-6-carbaldehyde (**252**), (4*S*)-4-(2-methylbutyl)-3-oxo-3,4-dihydro-1*H*-pyrrolo [2,1-c][1,4]oxazine-6-carbaldehyde (**253**), (*S*)-4-benzyl-3-oxo-3,4-dihydro-1*H*-pyrrolo [2,1-c][1,4]oxazine-6-carbaldehyde (**254**), flazin (**255**), perlolyrine (**256**), 1-hydroxy-β-carboline (**257**), lumichrome (**258**), 1*H*-indole-3-carboxaldehyde (**259**), [14,15],2-hydroxy-1-(1*H*-indol-3-yl), ethenone (**260**), and 5-(methoxymethyl)-1*H*-pyrrole-2-carbaldehyde (**261**).

**Figure 11:**
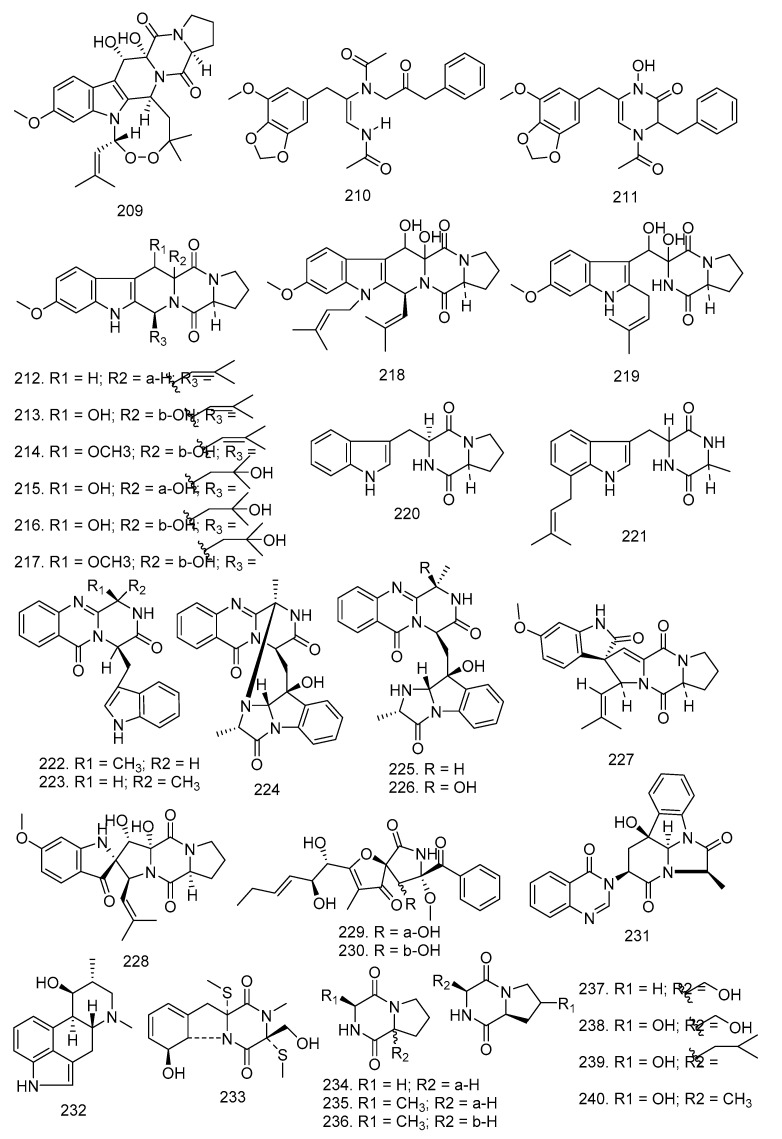

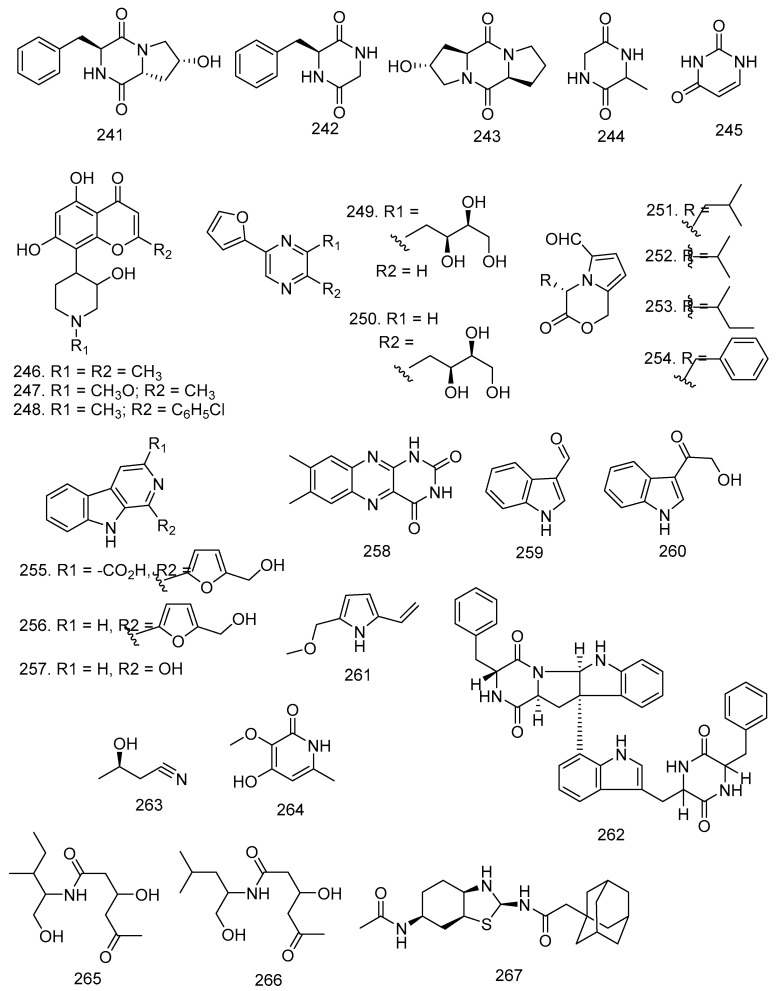
Alkaloids obtained from endophytic fungi in Meliaceae.

Asperazine (**262**) was discovered by Xiao et al. [44] from *Aspergillus* sp. KJ-9 in the stem bark of *M. azedarach.* Similar endophytic fungi and plants obtained (*R*)-3-hydroxybutanonitrile (**263**), 3-hydroxy-2-methoxy-5-methylpyridine-2(1*H*)-one (**264**), 3-hydroxy-*N*-(1-hydroxy-3-methylpentan-2-yl)-5-oxohexanamide (**265**), and 3-hydroxy-*N*-(1-hydroxy-4-methylpentan-2-yl)-5-oxohexanamide (**266**) [34]. In 2016, 2*S*,3a*R*,6*S*,7a*S*)-6-acetamido-octahydro-1,3-benzothiazole-2-yl-2-(adamantan-1-yl)acetamide (**267**) was discovered by Mittal et al. This compound (**267**) was isolated from *Emericella* sp. derived from *A. indica* twig [63], and the structures can be seen in Figure 11.

### 3.12. Nitro Compound, Fatty Acid, and Sugars

**Figure 12:**
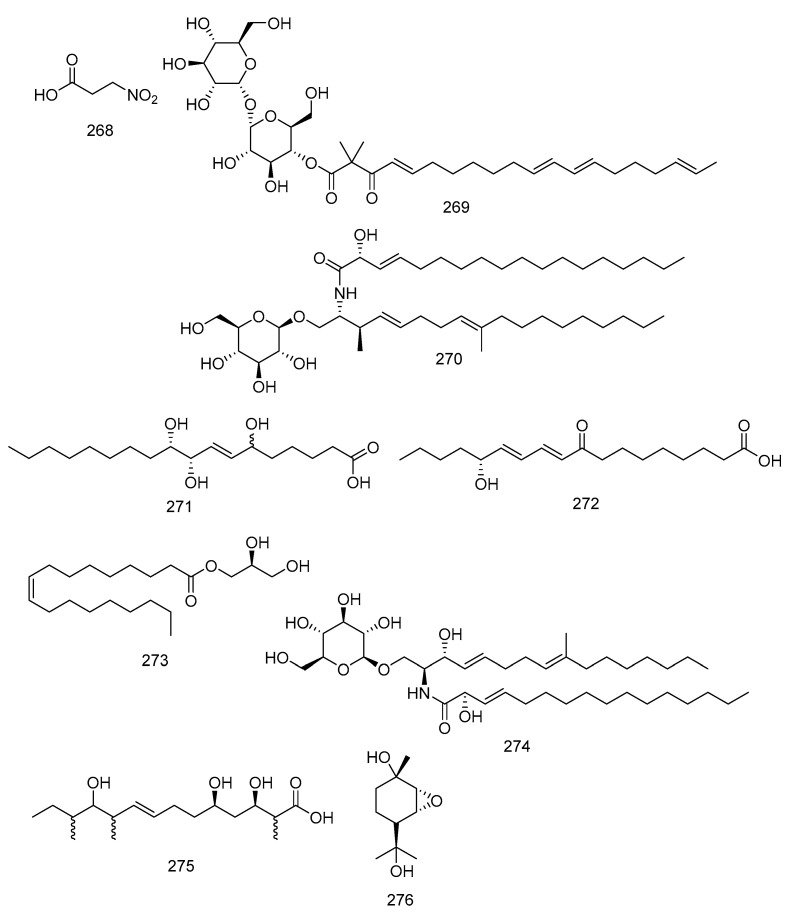
Nitro compound (**268**), fatty acid, and sugars (**269**–**276**) characterized by endophytic fungi in Meliaceae.

A nitro compound was isolated by Flores et al. [28] under the name 3-nitropropionic acid (**268**) from *P. longicolla* FJ 2759 fungi in the leaves of *T. elegans* A. Juss ssp. elegans. Furthermore, fatty acid and sugar were also obtained from endophytic fungi in Meliaceae. Fusaroside (**269**) was isolated from the organic extract of fermentation broths of an endophytic fungus, *Fusarium* sp. LN-11, in the leaves of *M. azedarach*. It is a unique trehalose-containing glycolipid composed of the 4-hydroxyl group of a trehalose unit attached to the carboxylic carbon of a long-chain fatty acid. Compound **269** was isolated with phalluside (**270**), (9*R*,10*R*,7*E*)-6,9,10-trihydroxyoctadec-7-enoic acid (**271**), porrigenic acid (**272**), and (9*Z*)-2,3-dihydroxypropyl octadeca-9-enoate (**273**) [21] (Figure 12).

The bark of this species also contained the fungus *B. dothidea* KJ-1, which was cultured in rice and produced cerebroside C (**274**) [34]. *D. eucalyptorum* KY-9 produced two biosynthetically related new metabolites, eucalyptacid A (**275**) and phomopene (**276**), from the different fungus (Figure 12) [50].

## 4. Bioactivity of Secondary Metabolites Isolated from Endophytic Fungi

### 4.1. Antimicrobial

Antimicrobial activity has been discovered in several compounds isolated from endophytic fungi. This activity provides antibiotics against pathogens and microorganisms such as *C. gloeosporioides*, *E. coli*, *B. subtilis*, *S. aureus*, and *B. cereus* that can cause food defects. Furthermore, antimicrobial is divided into antifungal and antibacterial [76,77,78,79,80,81,82]. Helvolic acid (**4**) was tested against fungi such as *B. cinerea*, *A. solani*, *A. alternata* (Fries) Keissler, *C. gloeosporioides*, *F. solani*, *F. oxysporum* f. sp. niveum, *F. oxysporum* f. sp. vasinfectum, and *G. saubinettii*, and compared with two positive controls, carbendazim and hymexazole. After analyzing the comparison, helvolic acid (**4**) was found to be active against the fungi *B. cinerea*, *A. solani*, *C. gloeosporioides*, *F. oxysporum* f. sp. niveum, and *G. saubinettii*. This compound can be an antifungal [32] (Table S2).

In other studies, pycnophorin (**7**) was evaluated against several fungi, *B. cinerea*, *A. solani*, *C. gloeosporioides*, and *G. saubinettii*, and active against *B. cinerea* and *A. solani*. This compound (**7**) was also evaluated for several bacteria, such as *E. coli*, *B. subtilis*, *S. aureus*, and *B. cereus*. However, these compounds were not active against the bacteria (Table S2) [34].

Wu et al. [35] reported that compounds **8**–**19** had been tested for their activity against several fungi, including *C. albicans YM 2005*, *A. niger YM 3029*, *P. oryzae YM 3051*, *F. avenaceum YM 3065*, and *H. compactum YM 3077*, with a positive control of nystatin. The results showed that nine sesquiterpenes (**8**–**16**) were moderately active against *C. albicans and H. compactum*, with MIC values ranging from 32 to 256 µg/mL. Meanwhile, diterpenes (**17**–**19**) were more active, with one exhibiting the most potent inhibitory activity against *C. albicans* and *P. oryzae*, with MIC values of 16 µg/mL [35]. In 2015, Huang et al. [36] continued the research conducted by Wu et al. and evaluated five new guaiane sesquiterpenes (**20**–**24**) against the same fungi as Wu et al. The guaiane sesquiterpenes showed moderate or weak antifungal activities in a broth microdilution assay.

Seven new phenolic bisabolane sesquiterpenoids (**47**–**53**) and known analogs (**54**–**58**) were evaluated by Pan et al. [40] against *S. aureus* ATCC 25923. The results showed that the compounds **48**, **49**, **51**, **53**, **55**, **57**, and **58** have moderate antibacterial activity against *S. aureus* ATCC 25923 with IC_50_ values in the range of 31.5–41.9 μM. Compound **68** was tested against the fungi *B. cinerea* and *A. solani*, which showed strong to moderate antifungal activities against *A. solani* (MIC of 6.25 µM) and did not have effects against *B. cinerea* [34]. Minimum Inhibitory Concentration (MIC) is the lowest concentration of a compound, usually a drug, which prevents visible growth of a bacterium or bacteria or pathogenic fungus. Compound **71** was tested against the bacteria *M. tenuis* (Order Micrococcales, Micrococcaceae) with a MIC value of 28.2 ± 0.52 µM, while **73** has antibacterial activity against *S. aureus* with a MIC value of 14.6 ± 0.47 µM [45]. In the study conducted by Geris dos Santos and Rodrigues-Fo [46], preaustinoids A-B (**78**–**79**) had a bacteriostatic effect on *S. aureus*, *P. aeruginosa*, *Bacillus* sp. at a dosage of 250 µg/mL and on *E. coli* at 125 µg. Furthermore, there was a bactericidal effect on *E. coli*, *P. aeruginosa*, and *Bacillus* sp. at dosages of 250 µg/mL with the control such as penicillin, vancomycin, and tetracycline tested at a conc. of 25 µg/mL.

Dianhydro-aurasperone C (**85**), isoaurasperone A (**86**), fonsecinone A (**87**), asperpyrone A (**88**), and rubrofusarin B (**89**) have been evaluated as antifungals against *G. saubinetti*, *M. grisea*, *B. cinerea*, *C. gloeosporioides*, and *A. solani.* The antibacterial activity against *B. cereus*, *S. aureus*, *B. subtilis*, and *E. coli* was also evaluated. The results showed that compound **87** was active against almost all phytopathogenic fungi tested with a minimum inhibitory concentration (MIC) range of 6.25–50 µM. Compound **87** was active against all pathogenic bacteria with MIC in the range of 25–100 µM [44]. Eucalactam B (**94**) was tested on several fungi, such as *A. solani*, *B. cinerea*, *F. solani*, and *G. saubinett*, and was not active [50].

Citrinin (**95**) has a bacteriostatic effect on *P. aeruginosa* and *B. subtilis* at 62.50 and 31.25 mg/mL. It has a bactericidal effect on *E. coli* and *P. aeruginosa* at 500 and 125 mg/mL dosages, respectively. Citrinin doses of 40 g/mL suppress *Leishmania mexicana* (order Trypanosomatide, Trypanosomatidae) growth following a 48-hour inoculation [51]. Furthermore, four new 10-membered lactones (**96**–**99**) and one known one (**100**) have been evaluated against *A. niger* YM 3029, *B. cinerea* YM 3061, *F. avenaceum* YM 3065, *F. moniliforme* YM 3067, *H. maydi*s YM 3076, *P. islandicum* YM 3104, and *O. minus* YM 3429 with control nystatin. Compound 99 demonstrated antifungal activity in the MIC value range of 31.25–500 µg/mL [26]. In 2009 Wu et al. continued testing the antifungal activity of lactone compounds (**101**–**102**) against *A. niger* YM 3029, *B. cinerea* YM 3061, *P. islandicum* YM 3104, and *O. minus* YM 3429. Similar to lactones (compounds **104**–**106**), Wu et al. [52] also evaluated several fungi such as *A. niger* YM 3029, *B. cinerea* YM 3061, *P. islandicum* YM 3104, and *O. minus*.

The javanicin (**110**) was inactive or only slightly active against fungi such as *Pythium ultimum, Phytophthora infestans, Botrytis cinerea*, and *Ceratocystis ulmi* but active against *Candida albicans, Escherichia coli, Bacillus* sp., and *Fusarium oxysporum* at higher MIC values ranging from 20 to 40 µg/mL [53]. Meanwhile, compound **111** was tested for its bacteriostatic effect on *P. aeruginosa* and *B. subtilis* at 7.81 and 31.25 mg/mL, respectively. The bactericidal effect on *E. coli, P. aeruginosa*, and *B. subtilis* at 500, 62.50, and 250 mg/mL, respectively, was also analyzed. Compound **113** has no bacteriostatic effect on *E. coli* and *B. subtilis* at a dosage of 500 mg/mL and also has no bactericidal effect on *P. aeruginosa* at 500 mg/mL. Compound **111** was almost completely inactive against *E. coli* but showed promising activity against *P. aeruginosa* and *B. subtilis* [51].

Zhang et al. [24] evaluated compounds **158**–**162** against the fungi *A. solani, B. cinerea, F. solani*, and *G. saubinettii*. Only **159** demonstrated a strong fungicidal effect (MIC of 12.5 µM) against *G. saubinetti* compared to the positive control hexanol (MIC of 25 µM). Other compounds demonstrated relatively poor properties, with MIC values greater than 50 µM against the pathogens tested. Compound **163** has been evaluated against *B. cinerea* (200 µM), *A. solani* (12.5 µM), *C. gloeosporioides* (200 µM), and *G. saubinettii* (>200 µM), compared to the control carbendazim: 12.5, 1.57, 1.57, 6.25 µM; hymexazol: 200, 6.25, >200, >200 µM; toosendanin: 200, 6.25, 200, 200 µM. In addition, compound 163 has activity against *A. solani* [34].

Compounds **183**–**185** have been tested against several fungi such as *B. cinerea, A. solani, A. alternata, C. gloeosporioides, F. solani, F. oxysporum* f. sp. niveum, *F. oxysporum* f. sp. vasinfectum, *G. saubinettii*. From the three compounds, **183** only showed good activity against the tested fungi, namely *B. cinerea* (12.5 µM), *A. solani* (12.5 µM), *A. alternata* (12.5 µM), *C. gloeosporioides* (12.5 µM), *F. solani* (50 µM), *F. oxysporum* f. sp. niveum (25 µM), *F. oxysporum* f. sp. *vasinfectum* (50 µM), and *G. saubinettii* (12.5 µM) [32]. Compounds **188** and **189** were tested against the same fungi, *B. cinerea* and *A. solani*. The results showed that **188** had activity against both fungi, while **189** affected only *A. solani* [34].

Compounds **190** and **192** were tested against the fungi *B. cinerea*, *A. solani, C. gloeosporioides,* and *G. saubinettii*. They showed no activity as well as compound **190**, which was tested against *E. coli*, *B. subtilis*, *S. aureus*, *B. cereus* with MIC results >100 µM [34]. Compounds **197**–**200** isolated by Gao et al. [50] were tested against several fungi *A. solani, B. cinerea, F. solani*, and *G. saubinetti*. The results were moderate to weak, but some active compounds, such as **198** and **200**, were active against *Alternaria solani* (12.5 and 6.25 µM). Compounds **209** and **212**–**245** were isolated by Li et al. [32] and evaluated against several fungi. Furthermore, sixteen compounds demonstrated potent antifungal activity against phytopathogenic fungi (*B. cinerea*, *A. solani*, *A. alternata*, *C. gloeosporioides*, *F. solani, F. oxysporum* f. sp. niveum, *F. oxysporum* f. sp. vasinfectum, and *G. saubinettii*). 12β-hydroxy-13α-methoxyverruculogen TR-2 (**217**), fumitremorgin B (**218**), and verruculogen (**209**) demonstrated antifungal activities with MIC values of 6.25−50 μg/mL, which were comparable to the two positive controls carbendazim and hymexazol, as seen in Table S2.

Compound **262** was tested against fungi and bacteria, each giving the following MIC values: *G. saubinetti*, MIC 25 M; *M. grisea*, MIC NA; *B. cinerea*, MIC 50 µM; *C. gloeosporioides*, MIC NA. It showed no activity against *B. cereus*; *S. aureus*, MIC 50 µM; *B. subtilis*, MIC NA; *E. coli*, MIC NA. Likewise, compound **263** gave the following MIC values: *G. saubinetti*, MIC 12.5 µM; *M. grisea*, MIC 25 µM; *B. cinerea*, MIC NA; *C. gloeosporioides*, MIC 50 µM; and *A. solani*, MIC 25 µM [44]. Eucalyptacid A (**275**) showed good activity against *A. solani* (12.5 µM), *B. cinerea* (50.0 µM), *F. solani* (25.0 µM), and *G. saubinetti* (50.0 µM), compared to positive control hymexazol (MIC 6.25, 50.0, 50.0, 25.0 µM, respectively) [50].

### 4.2. Cytotoxic Activity

Research on cytotoxic activity in endophytic fungi has been widely reported in many compounds with different test methods. More than 140 natural products with varying levels of antitumor activity have been isolated from fungal endophytes. Alkaloids, terpenes, steroids, polyketides, quinones, isocoumarins, esters, and other secondary metabolites are prevalent. The findings can be used to develop new antitumor drugs and endophyte resources [83,84,85,86,87]. Based on the study conducted by Chokpaibon et al., [31] sesquiterpenoid compounds merulin A-C (**1**–**3**) were analyzed for their cytotoxic activity against BT474 and SW620. Compound **3** had the highest activity and was continued by **1** and **2**. Compared with positive control, doxorubicin with respective IC_50_ values of 0.53 and 0.09 µg/mL had moderate activity.

Merulinols C-D (**37**–**38**) showed moderate activity against KATO-3 cells with IC_50_ values of 35.0 ± 1.20 µM and 25.3 ± 0.82 µM, respectively.[39]. Penicieduesmol B (**61**) has been tested against K-562 with an IC_50_ value of 90.1 µM (paclitaxel IC_50_ = 9.5 µM) [25]. Ergosterol peroxide (**67**) and *β*-sitosterol glucoside (**68**) were evaluated against the human colorectal HCT 116 cell line, and the results showed that the compound **67** was active with moderate activity IC_50_ value of 72.3 µM compared to positive control etoposide IC_50_ of 2.13 µM while **68** was not active [34,43]. Meanwhile, stemphyperylenol (**90**) indicated good activity against the HCT 116 cell line with an IC_50_ value of 3.13 µM, and the control was an etoposide with an IC_50_ of 2.13 µM [34]. Phomoxanthones G-K (**127**–**131**) were evaluated for their cytotoxicity against eight human tumor cell lines (A375, AGS, HCT-8, HCT-8/T, A549, MDA-MB-231, SMMC-7721, and A2780) using the MTT method with cisplatin as the positive control. However, none of them exhibited significant activity at 50 μM [62].

Chaetoglobosin C (**163**) and Chaetoglobosin F (164) were evaluated against the same cell line with an IC_50_ value of >100 and 26.5 µM, respectively. According to Luo et al., [59] phomopsichalasins D-G (**165**–**168**), 4′-hydroxy-deacetyl-18-deoxycytochalasin H (**169**), deacetyl-18-deoxycytochalasin H (**170**), 18-deoxycytochalasin H (**171**), cytochalasin H (**172**), deacetylcytochalasin H (**173**), and epoxycytochalasin H (**174**) were evaluated against several cells, as seen in Table S3.

Cytochalasin D (**175**) has cytotoxic activity against mouse lymphoma L5178Y cells with an EC_50_ value of 0.2 µM [72]. Compounds **186**–**193** were evaluated against the human colorectal HCT 116 cell line. The results showed that the compounds which have good-moderate activity after being compared to positive control etoposide IC_50_ value of 2.13 µM were **186** (IC_50_ 3.13 µM), **187** (IC_50_ 28.9 µM), **189** (IC_50_ 33.9 µM), and **190 (**IC_50_ 73.4 µM**)** [34]**.** Compounds **256**–**259** have been tested against Madin-Darby canine kidney (MDCK) normal cells (MTT assay) with IC_50_ values of 116.3 ± 12.1 µg/mL (**256**), 403.2 ± 31.4 µg/mL (**257**), 124.1 ± 10.5 µg/mL (**258**), and 522.5 ± 24.5 µg/mL (**259**). The positive control was ribavirin with an IC_50_ of 744.2 ± 18.5 µg/mL [62]. Compound **274** was tested but showed no activity against the human colorectal HCT 116 cell line [34].

### 4.3. Antioxidant and α-Glucoside Inhibitory Activity

Natural compounds isolated from endophytic fungi in medicinal plants are a rich source of drugs with various biological activities, including antioxidant properties. According to Kharat and Mendhulkar [88], phenolic compounds are responsible for antioxidant activity. Their presence may also have contributed to the radical scavenging activity observed in this study [88,89,90,91,92,93].

Wang et al., [40] reported the antioxidant activity of (−)-(7*S*)-10-hydroxysydonic acid (**58**) based on antioxidative activity to scavenge DPPH radicals with an IC_50_ value of 72.1 µM. Pycnophorin (**7**) and 3-hydroxy-2-methoxy-5-methylpyridin-2(1*H*)-one (**264**) were tested for DPPH radical scavenging with rates of 30.9% and 22.5% at conc. of 50 µM [34]. Furthermore, altenusin (**187**) and 5′-methoxy-6-methylbiphenyl-3,4,3′-triol (**190**) have DPPH radical scavenging activity with IC_50_ of 17.6 ± 0.23 and 18.7 ± 0.18 µM, respectively [34].

In several studies, the antioxidant activity test of isocoumarin compounds (**135**–**143**) showed two new isocoumarins, penicimarins L-N (**135**–**136**) with IC_50_ 28.3 and 38.9 µM, with seven known isocoumarins, peniisocoumarin E (**137**) (21.5 µM), apergilumarin A (**138**) (40.5 µM), penicimarin I (**139**) (IC_50_ > 50 µM), peniisocoumarin F (**140**) (IC_50_ > 50 µM), penicilloxalone B (**141**) (IC_50_ 30.3 µM), penicimarin G (**142**) (4.6 µM), and penicimarin H (**143**) (18.6 µM). In addition to antioxidant activity, isocoumarins were also evaluated as *α*-glucoside inhibitory activity and showed that compounds 139, 142, and 143 have activity with IC_50_ values of 776.5, 683.7, and 868.7 µM, respectively. Meanwhile, the control used was acarbose with IC_50_ of 313.9 µM [56].

### 4.4. Anti-Inflammatory and Anti-Influenza

Inflammation is a condition in which catabolism takes precedence over anabolism. It can also be defined as a defense mechanism that aids in the elimination of potentially harmful factors and establishes homeostasis in the body. This causes increased blood flow to the site of inflammation due to the increased permeability of capillaries and white blood cells, resulting in symptoms such as redness, swelling, and pain [94,95]. Endophytic fungi are a valuable source of pharmacologically active metabolites, one of which is anti-inflammatory [96,97]. Liu et al. [38] reported that pestaloporinate B (**28**) had anti-inflammatory activity (no inhibition in LpS-induced RAW 264.7 macrophage cells) and IC_50_ of 19.0 µM (positive control _L_-NMMA IC_50_ = 40.5 µM).

The influenza virus is one of the most common respiratory tract pathogenic agents, causing significant mortality, morbidity, and economic loss [98]. Alkaloids contain many important chemical compounds used to develop new anti-influenza agents [99]. The alkaloid group is one of the largest groups isolated from endophytic fungi [100]. In this study, 59 alkaloids were isolated. Wang et al. [62] evaluated alkaloid compounds (**249**–**261**) against the H_1_N_1_ virus. The compounds **257**–**259** were active against the influenza A virus subtype H_1_N_1_ with IC_50_ and selectivity index (SI) values of 38.3 (±1.2)/25.0 (±3.6)/39.7 (±5.6)/45.9 (±2.1) μg/mL and 3.0/16.1/3.1/11.4, respectively. The IC_50_ and SI values of the positive control, ribavirin, were 23.1 (±1.7) μg/mL and 32.2, respectively.

### 4.5. Brine Shrimp Lethality Test (BSLT)

The Brine Shrimp Lethality Test (BSLT) determines the bioactivity of a compound derived from natural ingredients. *Artemia salina* (order Anostraca, Artemilidae) from larvae is widely used in environmental studies, toxicity testing, and screening of bioactive compounds from plant extracts [101]. Helvetic acid (**4**) had activity when tested with BSLT and showed median lethal concentration LC_50_ of 73.7 µg/mL with the positive control toosendanin LC_50_ < 1 µg/mL [32]. Pycnophorin (**7**) and ergosterol peroxide (**67**) showed 43.81% and 13.72% lethality at conc. of 100 µM [24].

Isocoumarin and resorcylic acids were evaluated against brine shrimp (*Artemia salina*) with mortality rates of 78.2% for fusariumin (**144**) at concentration 10 µg/mL, 76.7% for aigialomycin D (**145**) at concentration 10 µg/mL and 82.8% for pochonin N (**146**) at conc. 10 µg/m [57]. Zhang et al. [24] discovered cytochalasin E (**162**), which was evaluated against *Artemia salina* and showed good activity with LC_50_ of 2.79 µM and 100% lethality at conc. of 50 µM. Meanwhile, chaetoglobosin F (**164**) showed 16.76% lethality at conc. of 100 µM.

Compounds **183**–**185** were evaluated against brine shrimp, and **183** had the best activity compared to **184** and **185** [24]**.** Compound **200** showed 36% lethality at conc of 100 µM. Conversely, compounds **209** and **212**–**245** were evaluated, and of the 18 compounds that showed moderate lethality in brine shrimps, **218** and **209** had significant toxicities, with LC_50_ values of 13.6 and 15.8 g/mL, respectively [32]. Based on Yang et al. [21], **269**–**273** showed growth inhibitory activity against brine shrimp with mortality rates of 47.6%, 64.8%, 26.2%, 20.9%, and 18.7% at conc. 10 µg/mL and 78.2% for the positive control fusariumin. Meanwhile, compound **276** showed 36% lethality at conc. of 100 µM [50].

### 4.6. Allelophatic Effects on Wheat Triticum Aestivum

Allelopathy is defined as a direct or indirect harmful or beneficial effect of one plant on another through chemical compounds released into the environment [102]. It has been used as a weed control strategy for commercial herbicide-dominated programs. One of the bioactivities of endophytic fungi compounds studied was allelophatic effects on wheat *Triticum aestivum* (order poales, poaceae) conducted by Rawat et al. [103]. Compounds **64, 162**, and **177**–**182** were evaluated for allelophatic effects on wheat *Triticum aestivum*. To some extent, all tested compounds inhibited *T. aestivum* shoot and root elongation with root intensity (RI) values ranging from 0.02 to 0.87 at 6.25 and 100 µM, respectively. The RI value is used as a direction to know the root growth of the individual species and for the sequence analysis. Furthermore, **179**, **64**, and **182** inhibited shoot elongation strongly, with IC_50_ values of 18.92 0.80, 23.58 0.43, and 24.02 0.51 µM, respectively. Compounds **180**, **182**, and **162** inhibited root elongation, with IC_50_ values of 17.35 0.05, 22.58 0.58, and 19.74 0.09 µM, respectively. These compounds were tested and compared with glyphosate, a commercial herbicide used as a positive control.

### 4.7. Antifeedant

Plant diseases caused by phytopathogens and pests cause crop loss, having a significant social and economic impact on the livelihoods of people relying on agricultural income. One of the ways to decrease the problem is to obtain antifeedant compounds. Endophytic fungi produced many compounds studied as antifeedants [104,105,106]. Li et al. studied antifungal, BSLT, and antifeedant activity in the previous study. Compounds **4**, **209**, **212**, **218**, **222**, **224**, **225**, **226**, and **228** were tested against armyworm (*Mythimna separata*; order Lepidoptera, Noctuidae), and each gave antifeedant index values of 7.5%, 55.0%, 15.0%, 30.0%, 10.0%, 10.0%, 45.0%, 7.5%, and 5.0% with the positive control toosendanin 97.5%. All compounds showed lower antifeedant rates than toosendanin [32].

### 4.8. Neuroprotective Activity

Neuroprotective activity is an action to save or regenerate the nervous system, cells, structures, and functions by preventing damage to the nervous system in neurochemical modulators. Neurological disabilities include a wide range of disorders, such as epilepsy, autism, neuromuscular disorders, brain tumors, cerebral palsy, Parkinson’s, and learning disabilities. Previous studies reported that polyketide compounds phomopsol A (**91**) and B (**92**) were tested on pC12 cells that cause Parkinson*’*s disease and respectively showed their cell viability results of 76% and 96% at conc. of 40.0 µM with positive control corticosterone 60% at 200.0 µM [55].

### 4.9. Anti-HIV Activity

Compounds phomoxanthones F, G, H, and K were tested in vitro by HIV-I virus-transfected 293 T cells. The result showed that phomoxanthone F at the concentration of 20 μM showed a weak inhibitory rate of 16.48 ± 6.67% with the positive control efavirenz, and an inhibitory rate of 88.54 ± 0.45% at the same concentration.

### 4.10. Phytotoxic Activity

The phytotoxic potential of plants and their compounds on other plants is increasingly being studied as a possible alternative to synthetic herbicides for controlling weeds in cropland. Apart from being an antifeedant, compounds from endophytic fungi were also tested for their phytotoxic activity such as compounds **148**, **150**, **153**, and **162** against *Digitaria ciliaris*, with the most significant result being compound **150**.

### 4.11. Enhanced Root Elongation Activity

The activity of a compound can be influenced by the surrounding environment. Compounds **152**, **153**, **154**, **156**, and **157** were tested for their activity on root elongation and the results showed that all the tested compounds increased root elongation activity. This is enough to warrant further studies on their mode of action and their role in chemical ecology, including allelopathy.

## 5. Conclusions

Meliaceae plant endophytes have been studied thus far for secondary metabolites. All endophytes use extra-cellular hydrolysis to defend against host attacks, invade pathogens, or obtain the host’s nutrients. Previous studies found that several of the same compounds were isolated from the host and its endophytes. Endophytic fungi are one of the microorganisms frequently found in plants. A comprehensive review of secondary metabolites (triterpenoids, sesquiterpenoids, alkaloids, flavonoids, quinones, and other compounds) isolated from Meliaceae endophytes and their pharmacological activities has provided information about species identification, compound isolation, and their vital role in medicine, which can be seen from their pharmacological activity. Endophytic fungi isolated from Meliaceae contain secondary metabolites with antioxidant, antibacterial, antifungal, anti-influenza, cytotoxic, and BSLT toxicity properties. This review provides sufficient evidence that endophytic fungi are potential bioactive plants. Hopefully, this review will contribute to the advancement of endophytic research, particularly in plants of the Meliaceae family.

## Data Availability

This study did not report any data.

## Supplementary Materials

The following supporting information can be downloaded at: https://www.mdpi.com/article/10.3390/molecules28020778/s1, Table S1: Secondary Metabolites Derived from Endophytic fungi-Meliaceae; Table S2: Antimicrobial Activity of Compounds that have been evaluated against several fungi and bacteria; Table S3: Cytotoxic Activity of compounds (**165**–**174**) against Several Cells.
