# Supplementary material for: Phytochemistry and Biological Activities of Endophytic Fungi from the Meliaceae Family
Source: Molecules. 2023 Jan 12;28(2):778. doi: 10.3390/molecules28020778 (PMC9863112; doi:10.3390/molecules28020778)
Supplement: Supplementary file 1 [file molecules-28-00778-s001.zip › molecules-2098929-supplementary.pdf]

Review

Phytochemistry and Biological Activities of Endophytic Fungi from the Meliaceae Family

Yeni Mulyani [1,2], Siska Elisahbet Sinaga [3] and Unang Supratman [1,3,*]

[1] Department of Chemistry, Faculty of Mathematic and Natural Sciences, Universitas Padjadjaran, Sumedang 45363, Indonesia
[2] Department of Marine Science, Faculty of Fisheries and Marine Science, Universitas Padjadjaran, Sumedang 45363, Indonesia
[3] Central Laboratory, Universitas Padjadjaran, Sumedang 45363, Indonesia
* Correspondence: unang.supratman@unpad.ac.id; Tel.: +62-22-779-4391

Supplementary Materials

Table S1. Secondary Metabolites Derived from Endophytic fungi-Meliaceae
Table S2. Antimicrobial Activity of Compounds that have been evaluated against several fungi and bacteria
Table S3. Cytotoxic Activity of compounds (**165–174)** against Several Cells

Table S1. Secondary Metabolites Derived from Endophytic fungi-Meliaceae.

Compounds	Molecular Formula	Molecular Mass	Fungal Strains	Host Plant	Plant Parts	Fermentation Media	Ref.
**Terpenoids**							
Merulin A (**1**)	$C_{14}H_{22}O_4$	254.1518	XG8D	*X. granatum*	Leaves	Corn steep-containing medium (broth)	[31]
Merulin B (**2**)	$C_{15}H_{24}O_5$	284.1624	XG8D	*X. granatum*	Leaves	Corn steep-containing medium (broth)	[31]
Merulin C (**3**)	$C_{15}H_{22}O_5$	282.1467	XG8D	*X. granatum*	Leaves	Corn steep-containing medium (broth)	[31]
Helvolic acid (**4**)	$C_{33}H_{44}O_8$	568.3036	*A. fumigatus* LN-4	*M. azedarach*	Stem bark	PD liquid medium	[32]
Azadirachtin A (**5**)	$C_{35}H_{44}O_{16}$	720.714	*E. parvum*	*A. indica* A. Juss.	Seeds. Leaves, Stem/Twigs, Inner Bark, Roots	Sabouraud dextrose broth (SB)	[33]
Azadirachtin B (**6**)	$C_{33}H_{42}O_{14}$	662.681	*E. parvum*	*A. indica* A. Juss.	Seeds. Leaves, Stem/Twigs, Inner Bark, Roots	Sabouraud dextrose broth (SB)	[33]
Pycnophorin (**7**)	$C_{27}H_{40}O_4$	428.2927	*B. dothidea* KJ-1	*M. azedarach* L.	Stem	Rice	[34]
(1*S*,4*S*,5*R*,7*R*,10*R*,11*R*)-guaiane-5,10,11,12-tetraol (**8**)	$C_{15}H_{28}O_4$	272.1988	*Xylaria* sp. YM 311647	*A. indica* A Juss	Stem	PDA, PDB	[35]
(1*S*,4*S*,5*S*,7*R*,10*R*,11*S*)-guaiane-1,10,11,12-tetraol (**9**)	$C_{15}H_{28}O_4$	272.1988	*Xylaria* sp. YM 311647	*A. indica* A Juss	Stem	PDA, PDB	[35]
(1*S*,4*S*,5*R*,7*R*,10*R*,11*S*)-guaiane-5,10,11,12-tetraol (**10**)	$C_{15}H_{28}O_4$	272.1988	*Xylaria* sp. YM 311647	*A. indica* A Juss	Stem	PDA, PDB	[35]
(1*S*,4*S*,5*S*,7*R*,10*R*,11*R*)-guaiane-1,10,11,12-tetraol (**11**)	$C_{15}H_{28}O_4$	272.1988	*Xylaria* sp. YM 311647	*A. indica* A Juss	Stem	PDA, PDB	[35]
(1*R*,3*S*,4*R*,5*S*,7*R*,10*R*,11*S*)-guaiane-3,10,11,12-tetraol (**12**)	$C_{15}H_{28}O_5$	288.1937	*Xylaria* sp. YM 311647	*A. indica* A Juss	Stem	PDA, PDB	[35]
(1*R*,3*R*,4*R*,5*S*,7*R*,10*R*,11*R*)-guaiane-3,10,11,12-tetraol (**13**)	$C_{15}H_{28}O_4$	272.1988	*Xylaria* sp. YM 311647	*A. indica* A Juss	Stem	PDA, PDB	[35]
(1*R*,4*S*,5*S*,7*S*,9*R*,10*S*,11*R*)-guaiane-9,10,11,12-tetraol (**14**)	$C_{15}H_{28}O_4$	272.1988	*Xylaria* sp. YM 311647	*A. indica* A Juss	Stem	PDA, PDB	[35]
(1*R*,4*S*,5*S*,7*R*,10*R*,11*S*)-guaiane-10,11,12-triol (**15**)	$C_{15}H_{28}O_3$	256.2038	*Xylaria* sp. YM 311647	*A. indica* A Juss	Stem	PDA, PDB	[35]

(1*R*,4*S*,5*S*,7*R*,10*R*,11*R*)-guaiane-10,11,12-triol (**16**)	$C_{15}H_{28}O_3$	256.2038	*Xylaria* sp. YM 311647	*A. indica* A Juss	Stem	PDA, PDB	[35]
14α,16-epoxy-18-norisopimar-7-en-4α-ol (**17**)	$C_{19}H_{30}O_2$	290.2246	*Xylaria* sp. YM 311647	*A. indica* A Juss	Stem	PDA, PDB	[35]
16-*O*-sulfo-18-norisopimar-7-en-4α,16-diol (**18**)	$C_{19}H_{32}O_5S$	372.1970	*Xylaria* sp. YM 311647	*A. indica* A Juss	Stem	PDA, PDB	[35]
9-deoxy-hymatoxin A (**19**)	$C_{20}H_{30}O_6S$	398.1763	*Xylaria* sp. YM 311647	*A. indica* A Juss	Stem	PDA, PDB	[35]
guaiane-2,10,11,12-tetraol (**20**)	$C_{15}H_{28}O_4$	272.1988	*Xylaria* sp. YM 311647	*A. indica* A Juss	Stem	PDA, PDB	[36]
guaiane-2,4,10,11,12-pentaol (**21**)	$C_{15}H_{28}O_5$	288.1937	*Xylaria* sp. YM 311647	*A. indica* A Juss	Stem	PDA, PDB	[36]
guaiane-4,5,10,11,12-pentaol (**22**)	$C_{15}H_{28}O_5$	288.1937	*Xylaria* sp. YM 311647	*A. indica* A Juss	Stem	PDA, PDB	[36]
guaiane-1,5,10,11,12-pentaol (**23**)	$C_{15}H_{28}O_5$	288.1937	*Xylaria* sp. YM 311647	*A. indica* A Juss	Stem	PDA, PDB	[36]
11-methoxyguaiane-4,10,12-triol (**24**)	$C_{16}H_{30}O_4$	286.2144	*Xylaria* sp. YM 311647	*A. indica* A Juss	Stem	PDA, PDB	[36]
(9*R*, 10*R*)-dihydroharzianone (**25**)	$C_{20}H_{32}O$	288.2453	*Trichoderma* sp. Xy24	*X. granatum*	Leaves, Stems, and Peels	PDA liquid medium	[37]
Harzianelactone (**26**)	$C_{20}H_{30}O_2$	302.2246	*Trichoderma* sp. Xy24	*X. granatum*	Leaves, Stems, and Peels	PDA liquid medium	[37]
Pestaloporinate A (**27**)	$C_{21}H_{32}O_7$	396.2148	*Pestalotiopsis* sp.	*M. azaderach* Linn	Stem Bark	PDB	[38]
Pestaloporinate B (**28**)	$C_{19}H_{26}O_6$	350.1729	*Pestalotiopsis* sp.	*M. azaderach* Linn	Stem Bark	PDB	[38]
Pestaloporinate C (**29**)	$C_{18}H_{26}O_5$	322.1780	*Pestalotiopsis* sp.	*M. azaderach* Linn	Stem Bark	PDB	[38]
Pestaloporinate D (**30**)	$C_{18}H_{26}O_5$	322.1780	*Pestalotiopsis* sp.	*M. azaderach* Linn	Stem Bark	PDB	[38]
Pestaloporinate E (**31**)	$C_{20}H_{32}O_6$	368.2199	*Pestalotiopsis* sp.	*M. azaderach* Linn	Stem Bark	PDB	[38]
Pestaloporinate F (**32**)	$C_{18}H_{28}O_4$	308.1988	*Pestalotiopsis* sp.	*M. azaderach* Linn	Stem Bark	PDB	[38]
Pestaloporinate G (**33**)	$C_{19}H_{30}O_4$	322.2144	*Pestalotiopsis* sp.	*M. azaderach* Linn	Stem Bark	PDB	[38]
14-acetylhumulane (**34**)	$C_{16}H_{22}O_5$	294.1467	*Pestalotiopsis* sp.	*M. azaderach* Linn	Stem Bark	PDB	[38]

Merulinol A (**35**)	$C_{14}H_{22}O_4$	254.1518	XG8D	*X. granatum*	Leaves	Sabouraud Dextrose Broth (SDB)	[39]
Merulinol B (**36**)	$C_{15}H_{26}O_3$	254.1882	XG8D	*X. granatum*	Leaves	Sabouraud Dextrose Broth (SDB)	[39]
Merulinol C (**37**)	$C_{16}H_{26}O_3$	266.1882	XG8D	*X. granatum*	Leaves	Sabouraud Dextrose Broth (SDB)	[39]
Merulinol D (**38**)	$C_{16}H_{26}O_3$	266.1882	XG8D	*X. granatum*	Leaves	Sabouraud Dextrose Broth (SDB)	[39]
Merulinol E (**39**)	$C_{15}H_{20}O_4$	264.1362	XG8D	*X. granatum*	Leaves	Sabouraud Dextrose Broth (SDB)	[39]
Merulinol F (**40**)	$C_{15}H_{24}O_5$	284.1624	XG8D	*X. granatum*	Leaves	Sabouraud Dextrose Broth (SDB)	[39]
Aciicolinol C (**41**)	$C_{15}H_{24}O_4$	268.1675	XG8D	*X. granatum*	Leaves	Sabouraud Dextrose Broth (SDB)	[39]
Aciicolinol K (**42**)	$C_{15}H_{22}O_5$	282.1467	XG8D	*X. granatum*	Leaves	Sabouraud Dextrose Broth (SDB)	[39]
Aciicolinol F (**43**)	$C_{15}H_{24}O_3$	252.1725	XG8D	*X. granatum*	Leaves	Sabouraud Dextrose Broth (SDB)	[39]
Aciicolinol D (**44**)	$C_{15}H_{24}O_3$	252.1725	XG8D	*X. granatum*	Leaves	Sabouraud Dextrose Broth (SDB)	[39]
Colletotrin (**45**)	$C_{29}H_{42}O_9$	534.2829	*C. gloeosporioides*	*T. monadelpha*	Stem bark	Rice	[27]
Hydroheptelidic acid (**46**)	$C_{15}H_{22}O_5$	282.1467	*C. gloeosporioides*	*T. monadelpha*	Stem bark	Rice	[27]
(7*R*,10*S*)-7,10-epoxysydonic acid (**47**)	$C_{15}H_{20}O_4$	264.1362	*Aspergillus* sp. xy02	*X. moluccensis*	Leaves	Rice + Sea Salt	[40]
(7*S*,10*S*)-7,10-epoxysydonic acid (**48**)	$C_{15}H_{20}O_4$	264.1362	*Aspergillus* sp. xy02	*X. moluccensis*	Leaves	Rice + Sea Salt	[40]
(7*R*,11*S*)-7,12-epoxysydonic acid (**49**)	$C_{15}H_{20}O_4$	264.1362	*Aspergillus* sp. xy02	*X. moluccensis*	Leaves	Rice + Sea Salt	[40]
(7*S*,11*S*)-7,12-epoxysydonic acid (**50**)	$C_{15}H_{20}O_4$	264.1362	*Aspergillus* sp. xy02	*X. moluccensis*	Leaves	Rice + Sea Salt	[40]
7-deoxy-7,14-didehydro-12-hydroxysydonic acid (**51**)	$C_{15}H_{20}O_4$	264.1362	*Aspergillus* sp. xy02	*X. moluccensis*	Leaves	Rice + Sea Salt	[40]
(*Z*)-7-deoxy-7,8-didehydro-12-hydro-xysydonic acid (**52**)	$C_{15}H_{20}O_4$	264.1362	*Aspergillus* sp. xy02	*X. moluccensis*	Leaves	Rice + Sea Salt	[40]
(*E*)-7-deoxy-7,8-didehydro-12-hydroxysydonic acid (**53**)	$C_{15}H_{20}O_4$	264.1362	*Aspergillus* sp. xy02	*X. moluccensis*	Leaves	Rice + Sea Salt	[40]
(+)-1-hydroxyboivinianic acid (**54**)	$C_{12}H_{12}O_5$	236.0685	*Aspergillus* sp. xy02	*X. moluccensis*	Leaves	Rice + Sea Salt	[40]
engyodontiumone I (**55**)	$C_{15}H_{20}O_4$	264.1362	*Aspergillus* sp. xy02	*X. moluccensis*	Leaves	Rice + Sea Salt	[40]
(+)-sydonic acid (**56**)	$C_{15}H_{22}O_4$	266.1518	*Aspergillus* sp. xy02	*X. moluccensis*	Leaves	Rice + Sea Salt	[40]
(+)-hydroxysydonic acid (**57**)	$C_{15}H_{22}O_5$	282.1467	*Aspergillus* sp. xy02	*X. moluccensis*	Leaves	Rice + Sea Salt	[40]
(−)-(7*S*)-10-hydroxysydonic acid (**58**)	$C_{15}H_{22}O_5$	282.1467	*Aspergillus* sp. xy02	*X. moluccensis*	Leaves	Rice + Sea Salt	[40]

Guaidiol (**59**)	$C_{17}H_{30}O$	250.2297	*Xylaria* sp. HNWSW-2	*X. granatum*	Stem	Rice	[41]
Penicieudesmol A (**60**)	$C_{15}H_{26}O_2$	238.2933	*Penicillium* sp. J-54	*C. tagal*	Leaves	Potato and Glucose Broth	[25]
Penicieduesmol B (**61**)	$C_{15}H_{26}O_3$	254.1882	*Penicillium* sp. J-54	*C. tagal*	Leaves	Potato and Glucose Broth	[25]
Penicieudesmol C (**62**)	$C_{15}H_{26}O_3$	254.1882	*Penicillium* sp. J-54	*C. tagal*	Leaves	Potato and Glucose Broth	[25]
Penicieudesmol D (**63**)	$C_{15}H_{26}O_4$	270.1831	*Penicillium* sp. J-54	*C. tagal*	Leaves	Potato and Glucose Broth	[25]
Hydroxyldecandrin G (**64**)	$C_{20}H_{28}O_4$	332.1988	*Xylaria* sp. XC-16	*T. sinensis*	Leaves	PD liquid medium	[42]
**Steroids**							
Ergokonin B (**65**)	$C_{28}H_{42}O_5$	458.3032	*Fusarium* sp. LN-11	*M. azedarach*	Leaves	Liquid Culture	[21]
Cerevisterol (**66**)	$C_{28}H_{46}O_3$	430.3447	*Fusarium* sp. LN-11	*M. azedarach*	Leaves	Liquid Culture	[21]
			F. phaseoli	*C. macrophyllus*	Root	Unpolished brown rice	[43]
Ergosterol peroxide (**67**)	$C_{28}H_{44}O_3$	428.3290	*B. dothidea* KJ-1	*M. azedarach L.*	Stem	Rice	[44]
			F. phaseoli	*C. macrophyllus*	Root	Unpolished brown rice	[43]
β-sitosterol glucoside (**68**)	$C_{34}H_{58}O_6$	562.4233	*B. dothidea* KJ-1	*M. azedarach L.*	Stem	Rice	[44]
Ergosterol **(69)**	$C_{28}H_{44}O$	396.3392	*Eupenicillium* sp. HJ002	*X. granatum*		Potato Liquid Medium (33g sea salt in 1L of potato infusion)	[22]
			F. phaseoli	*C. macrophyllus*	Root	Unpolished brown rice	[22]
5β,6β-epoxy-3β,15α-dihydroxy-(22*E*,24*R*)-ergosta-8(14),22-dien-7-one (**70**)	$C_{28}H_{42}O_4$	442.3083	*Phomopsis* sp. MGF222	*X. granatum*		Potato Liquid Medium + Sea Salt	[45]
5β,6β-epoxy-3β,7α-dihydroxy(22*E*,24*R*)-ergosta-8(14),22-dien-15-one (**71**)	$C_{28}H_{42}O_4$	442.3083	*Phomopsis* sp. MGF222	*X. granatum*		Potato Liquid Medium + Sea Salt	[45]
5β,6β-epoxy-3β,7α,9α-trihydroxy-(22*E*,24*R*)-ergosta-8(14),22-dien-15-one (**72**)	$C_{28}H_{42}O_5$	458.3032	*Phomopsis* sp. MGF222	*X. granatum*		Potato Liquid Medium + Sea Salt	[45]
3β,9α,15α-trihydroxy-(22*E*,24*R*)-10(5→ 4) abeo-ergosta-6,8(14),22-trien-5-one (**73**)	$C_{28}H_{42}O_4$	442.3083	*Phomopsis* sp. MGF222	*X. granatum*		Potato Liquid Medium + Sea Salt	[45]
3,15-dihydroxyl-(22*E*,24*R*)-ergosta-5,8(14),22trien-7-one (**74**)	$C_{28}H_{42}O_3$	426.3134	*Phomopsis* sp. MGF222	*X. granatum*		Potato Liquid Medium + Sea Salt	[45]
(22*E*,24*R*)-ergosta-4,6,8(14),22-tetraen-3,15-dione (**75**)	$C_{28}H_{38}O_2$	406.2872	*Phomopsis* sp. MGF222	*X. granatum*		Potato Liquid Medium + Sea Salt	[45]

Ergost-5,22*E*-dien-3β-oleate-20-ol (**76**)	$C_{47}H_{80}O_3$	692.6107	*F. phaseoli*	*C. macrophyllus*	Root	Unpolished brown rice	[43]
Atroside (**77**)	$C_{51}H_{90}O_7$	814.6687	*F. phaseoli*	*C. macrophyllus*	Root	Unpolished brown rice	[43]
**Meroterpenes**							
Preaustinoid A (**78**)	$C_{26}H_{36}O_6$	444.2508	*Penicillium* sp.	*M. azedarach*	Root bark	Rice	[46]
Preaustinoid B (**79**)	$C_{26}H_{36}O_6$	444.2505	*Penicillium* sp.	*M. azedarach*	Root bark	Rice	[46]
Preaustinoid A1 (**80**)	$C_{26}H_{36}O_7$	460.2461	*P. brasilianum*	*M. azedarach*	Root bark	Rice	[47]
Preaustinoid B2 (**81**)	$C_{24}H_{34}O_5$	402.2406	*P. brasilianum*	*M. azedarach*	Root bark	Rice	[47]
Austinolide (**82**)	$C_{22}H_{26}O_9$	434.1577	*P. brasilianum*	*M. azedarach*	Root bark	Rice	[47]
**Polyketide**							
Aurasperone A (**83**)	$C_{32}H_{26}O_{10}$	570.1526	*A. aculeatus Lizuka*	*M. azedarach*		Parboiled Rice	[48]
Fonsecinone A (**84**)	$C_{32}H_{26}O_{10}$	570.1526	*A. aculeatus Lizuka*	*M. azedarach*		Parboiled Rice	[48]
Dianhydro-aurasperone C (**85**)	$C_{31}H_{24}O_{10}$	556.1369	*Aspergillus* sp. KJ-9	*M. azedarach*	Stem Bark		[44]
Isoaurasperone A (**86**)	$C_{32}H_{26}O_{10}$	570.1526	*Aspergillus* sp. KJ-9	*M. azedarach*	Stem Bark		[44]
Fonsecinone A (**87**)	$C_{31}H_{24}O_{10}$	556.1369	*Aspergillus* sp. KJ-9	*M. azedarach*	Stem Bark		[44]
Asperpyrone A (**88**)	$C_{31}H_{24}O_{10}$	556.1369	*Aspergillus* sp. KJ-9	*M. azedarach*	Stem Bark		[44]
Rubrofusarin B (**89**)	$C_{16}H_{14}O_5$	286.0841	*Aspergillus* sp. KJ-9	*M. azedarach*	Stem Bark		[44]
Stemphyperylenol (**90**)	$C_{20}H_{16}O_6$	392.0947	*B. dothidea* KJ-1	*M. azedarach* L.	Stem	Rice	[34]
Phomopsol A (**91**)	$C_{20}H_{19}NO_4$	337.1314	*Phomopsis* sp. xy21	*X. granatum*	Leaves		[49]
Phomopsol B (**92**)	$C_{16}H_{18}O_5$	290.1154	*Phomopsis* sp. xy21	*X. granatum*	Leaves		[49]
3-(2,6-dihydroxyphenyl)-4-hydroxy-6methylisobenzofuran-1(3H)-one (**93**)	$C_{15}H_{12}O_5$	272.0685	*Phomopsis* sp. xy21	*X. granatum*	Leaves		[49]
Eucalactam B (**94**)	$C_{24}H_{31}N_3O_8$	499.2894	*D. eucalyptorum* KY-9	*M. azedarach*	Leaves	Rice	[50]
Citrinin (**95**)	$C_{13}H_{14}O_5$	250.0841	*P. janthinellum*	*M. azedarach*	Fruit	White corn	[51]
**Lactone**							
8α-acetoxy-5α-hydroxy-7-oxodecan-10-olide (**96**)	$C_{12}H_{18}O_6$	258.1103	*Phomopsis* sp.	*A. indica*	Stem	PDB	[26]
7α,8α-dihydroxy-3,5-decadien-10-olide (**97**)	$C_{10}H_{14}O_4$	198.0892	*Phomopsis* sp.	*A. indica*	Stem	PDB	[26]

7α-acetoxymultiplolide A (**98**)	$C_{12}H_{16}O_6$	257.1032	*Phomopsis* sp.	*A. indica*	Stem	PDB	[26]
8α-acetoxymultiplolide A (**99**)	$C_{12}H_{16}O_6$	257.1021	*Phomopsis* sp.	*A. indica*	Stem	PDB	[26]
Multiplolide A (**100**)	$C_{10}H_{14}O_5$	214.0841	*Phomopsis* sp.	*A. indica*	Stem	PDB	[26]
Nigrosporalactone (**101**)	$C_8H_{12}O_3$	156.0786	*Nigrospora* sp. YB-141	*A. indica* A. Juss.	Stem	PDB	[52]
Phomalactone (**102**)	$C_8H_{10}O_3$	154.0630	*Nigrospora* sp. YB-141	*A. indica* A. Juss.	Stem	PDB	[52]
(*R*)-striatisporolide A (**103**)	$C_{11}H_{16}O_4$	212.1049	*Eupenicillium* sp. HJ002	*X. granatum*		Potato Liquid Medium (33g sea salt in 1L of potato infusion)	[22]
**Pyrone**							
Solanapyrone N (**104**)	$C_{18}H_{23}NO_4$	317.1627	*Nigrospora* sp. YB-141	*A. indica* A. Juss.	Stem	PDB	[52]
Solanapyrone O (**105**)	$C_{19}H_{25}NO_3$	315.1834	*Nigrospora* sp. YB-141	*A. indica* A. Juss.	Stem	PDB	[52]
Solanapyrone C (**106**)	$C_{19}H_{25}NO_4$	331.1784	*Nigrospora* sp. YB-141	*A. indica* A. Juss.	Stem	PDB	[52]
Astropyrone (**107**)	$C_{13}H_{18}O_5$	254.1154	*Xylaria* sp. HNWSW-2	*X. granatum*	Stem	Rice	[41]
Xylaropyrone B (**108**)	$C_{12}H_{18}O_4$	226.1205	*Xylaria* sp. HNWSW-2	*X. granatum*	Stem	Rice	[41]
Xylaropyrone C (**109**)	$C_{12}H_{18}O_4$	226.1205	*Xylaria* sp. HNWSW-2	*X. granatum*	Stem	Rice	[41]
**Quinone**							
Javanicin (**110**)	$C_{15}H_{14}O_6$	290.0790	*Chloridium* sp.	*A. indica*	Root	PDB	[53]
**Anthraquinone**							
Emodin (1,6,8-trihydroxy-3-methylanthraquinone) (**111**)	$C_{15}H_{10}O_5$	270.0528	*P. janthinellum*	*M. azedarach*	Fruit	White corn	[51]
Citreorosein (1,6,8-trihydroxy-3-hydroxymethylanthraquinone) (**112**)	$C_{15}H_{10}O_6$	286.0477	*P. janthinellum*	*M. azedarach*	Fruit	White corn	[51]
Janthinone (**113**)	$C_{16}H_{12}O_5$	284.0685	*P. janthinellum*	*M. azedarach*	Fruit	White corn	[51]
Arugosin O (**114**)	$C_{26}H_{30}O_5$	422.2093	*Xylariaceae* sp.	*L. domesticum*	Leaves	Rice	[54]
Arugosin P (**115**)	$C_{33}H_{42}O_8$	566.2880	*Xylariaceae* sp.	*L. domesticum*	Leaves	Rice	[54]
Arugosin Q (**116**)	$C_{32}H_{42}O_7$	538.2931	*Xylariaceae* sp.	*L. domesticum*	Leaves	Rice	[54]
Arugosin K (**117**)	$C_{22}H_{24}O_5$	368.1624	*Xylariaceae* sp.	*L. domesticum*	Leaves	Rice	[54]
Arugosin A (**118**)	$C_{25}H_{28}O_6$	424.1886	*Xylariaceae* sp.	*L. domesticum*	Leaves	Rice	[54]
Arugosin B (**119**)	$C_{25}H_{28}O_6$	424.1886	*Xylariaceae* sp.	*L. domesticum*	Leaves	Rice	[54]

Arugosin N (**120**)	$C_{20}H_{20}O_5$	340.1311	*Xylariaceae* sp.	*L. domesticum*	Leaves	Rice	[54]
1,6,10-trihydroxy-8-methyl-2-(3-methyl-2- butenyl)-dibenz[b,e]oxepin-11(6H)-one (**121**)	$C_{20}H_{20}O_5$	340.1311	*Xylariaceae* sp.	*L. domesticum*	Leaves	Rice	[54]
Arugosin L (**122**)	$C_{22}H_{24}O_5$	368.1624	*Xylariaceae* sp.	*L. domesticum*	Leaves	Rice	[54]
Arugosin M (**123**)	$C_{21}H_{22}O_5$	354.1467	*Xylariaceae* sp.	*L. domesticum*	Leaves	Rice	[54]
Arugosin F (**124**)	$C_{15}H_{12}O_5$	272.0685	*Xylariaceae* sp.	*L. domesticum*	Leaves	Rice	[54]
Arugosin G (**125**)	$C_{30}H_{36}O_6$	492.2512	*Xylariaceae* sp.	*L. domesticum*	Leaves	Rice	[54]
**Xanthone**							
Phomoxanthone F (**126**)	$C_{14}H_{12}O_6$	276.0634	*Phomopsis* sp. xy21	*X. granatum*	Leaves	Rice + Sea Salt	[55]
Phomoxanthone G (**127**)	$C_{15}H_{16}O_7$	308.0896	*Phomopsis* sp. xy21	*X. granatum*	Leaves	Rice + Sea Salt	[55]
Phomoxanthone H (**128**)	$C_{15}H_{16}O_7$	308.0896	*Phomopsis* sp. xy21	*X. granatum*	Leaves	Rice + Sea Salt	[55]
Phomoxanthone I (**129**)	$C_{15}H_{16}O_5$	276.0998	*Phomopsis* sp. xy21	*X. granatum*	Leaves	Rice + Sea Salt	[55]
Phomoxanthone J (**130**)	$C_{15}H_{12}O_6$	288.0634	*Phomopsis* sp. xy21	*X. granatum*	Leaves	Rice + Sea Salt	[55]
Phomoxanthone K (**131**)	$C_{15}H_{10}O_7$	302.0427	*Phomopsis* sp. xy21	*X. granatum*	Leaves	Rice + Sea Salt	[55]
Leptosphaerin E (**132**)	$C_{15}H_{12}O_5$	272.0685	*Phomopsis* sp. xy21	*X. granatum*	Leaves	Rice + Sea Salt	[55]
Mono-dictyxanthone (8-hydroxy-3-methyl-9-oxo-9H-xanthene-1-carboxylic acid) (**133**)	$C_{15}H_{10}O_5$	270.0528	*Phomopsis* sp. xy21	*X. granatum*	Leaves	Rice + Sea Salt	[55]
2,2′,6′-trihydroxy-4-methyl-6-methoxy-acyl-diphenylmethanone (**134**)	$C_{16}H_{14}O_6$	302.0790	*Phomopsis* sp. xy21	*X. granatum*	Leaves	Rice + Sea Salt	[55]
**Isocoumarin**							
Penicimarin L (**135**)	$C_{13}H_{14}O_6$	266.0790	*Penicillium* sp. MGP11	*X. granatum*	Root	70g rice with 100 mL seawater (33g sea salt in 1 L distilled water)	[56]
Penicimarin M (**136**)	$C_{15}H_{18}O_5$	278.1154	*Penicillium* sp. MGP11	*X. granatum*	Root	70g rice with 100 mL seawater (33g sea salt in 1 L distilled water)	[56]
Peniisocoumarin E (**137**)	$C_{14}H_{16}O_6$	280.0947	*Penicillium* sp. MGP11	*X. granatum*	Root	70g rice with 100 mL seawater (33g sea salt in 1 L distilled water)	[56]
Apergilumarin A (**138**)	$C_{14}H_{16}O_4$	248.1049	*Penicillium* sp. MGP11	*X. granatum*	Root	70g rice with 100 mL seawater (33g sea salt in 1 L distilled water)	[56]

Penicimarin I (**139**)	$C_{14}H_{16}O_5$	264.0998	*Penicillium* sp. MGP11	*X. granatum*	Root	70g rice with 100 mL seawater (33g sea salt in 1 L distilled water)	[56]
Peniisocoumarin F (**140**)	$C_{14}H_{18}O_5$	266,1154	*Penicillium* sp. MGP11	*X. granatum*	Root	70g rice with 100 mL seawater (33g sea salt in 1 L distilled water)	[56]
Penicilloxalone B (**141**)	$C_{14}H_{16}O_5$	264.0998	*Penicillium* sp. MGP11	*X. granatum*	Root	70g rice with 100 mL seawater (33g sea salt in 1 L distilled water)	[56]
Penicimarin G (**142**)	$C_{15}H_{20}O_5$	280.1311	*Penicillium* sp. MGP11	*X. granatum*	Root	70g rice with 100 mL seawater (33g sea salt in 1 L distilled water)	[56]
Penicimarin H (**143**)	$C_{15}H_{18}O_5$	278.1154	*Penicillium* sp. MGP11	*X. granatum*	Root	70g rice with 100 mL seawater (33g sea salt in 1 L distilled water)	[56]
Fusariumin (**144**)	$C_{18}H_{22}O_5$	318.1467	*Fusarium* sp. LN-10	*M. azedarach*	Leaves	PDB	[57]
**Resorcylic Acid**							
Aigialomycin D (**145**)	$C_{18}H_{22}O_6$	334.1416	*Fusarium* sp. LN-10	*M. azedarach*	Leaves	PDB	[57]
Pochonin N (**146**)	$C_{18}H_{27}ClO_7$	384.0967	*Fusarium* sp. LN-10	*M. azedarach*	Leaves	PDB	[57]
Zearalenone (**147**)	$C_{18}H_{22}O_5$	318.1467	*Fusarium* sp. LN-10	*M. azedarach*	Leaves	PDB	[57]
(15*S*)-de-O-methyllasiodiplodin (**148**)	$C_{16}H_{22}O_4$	278.1518	*L. theobromae* GC-22	*X. granatum*	Dead Branch	Unpolished Rice	[58]
(13*S*,15*S*)-13-hydroxy-de-*O*-methyllasiodiplodin (**149**)	$C_{16}H_{22}O_5$	294.1467	*L. theobromae* GC-22	*X. granatum*	Dead Branch	Unpolished Rice	[58]
(14*S*,15*S*)-14-hydroxy-de-*O*-methyllasiodiplodin (**150**)	$C_{16}H_{22}O_5$	294.1467	*L. theobromae* GC-22	*X. granatum*	Dead Branch	Unpolished Rice	[58]
(13*R*,14*S*,15*S*)-13,14-dihydroxy-de-*O*-methyllasiodiplodin (**151**)	$C_{16}H_{22}O_6$	310.1416	*L. theobromae* GC-22	*X. granatum*	Dead Branch	Unpolished Rice	[58]
ethyl (S)-2,4-dihydroxy-6-(8-hydroxynonyl)benzoate (**152**)	$C_{18}H_{28}O_5$	324.1937	*L. theobromae* GC-22	*X. granatum*	Dead Branch	Unpolished Rice	[58]
ethyl 2,4-dihydroxy-6-(8-hydroxyheptyl) benzoate (**153**)	$C_{16}H_{24}O_5$	296.1624	*L. theobromae* GC-22	*X. granatum*	Dead Branch	Unpolished Rice	[58]
ethyl 2,4-dihydroxy-6-(4-methoxycarbonylbutyl)benzoate (**154**)	$C_{15}H_{20}O_6$	296.1260	*L. theobromae* GC-22	*X. granatum*	Dead Branch	Unpolished Rice	[58]

3-(2-ethoxycarbonyl-3,5-dihydroxyphen- yl)propionic acid (**155**)	$C_{12}H_{14}O_6$	254.0790	*L. theobromae* GC-22	*X. granatum*	Dead Branch	Unpolished Rice	[58]
(S)-2,4-dihydroxy-6-(8-hydroxynonyl)benzoate (**156**)	$C_{20}H_{32}O_5$	352.2250	*L. theobromae* GC-22	*X. granatum*	Dead Branch	Unpolished Rice	[58]
ethyl 2,4-dihydroxy-6-(8-oxononyl)benzoate (**157**)	$C_{18}H_{26}O_5$	322.1780	*L. theobromae* GC-22	*X. granatum*	Dead Branch	Unpolished Rice	[58]
**Cytochalasans**							
Cytochalasin Z_{27} (**158**)	$C_{28}H_{33}NO_6$	479.2308	*Xylaria* sp. XC-16	*T. sinensis*	Leaves	Rice	[24]
Cytochalasin Z_{28} (**159**)	$C_{28}H_{33}NO_6$	479.2308	*Xylaria* sp. XC-16	*T. sinensis*	Leaves	Rice	[24]
Seco-cytochalasin E (**160**)	$C_{29}H_{37}NO_8$	527.2519	*Xylaria* sp. XC-16	*T. sinensis*	Leaves	Rice	[24]
Cytochalasin Z_{18} (**161**)	$C_{32}H_{45}NO_8$	571.3145	*Xylaria* sp. XC-16	*T. sinensis*	Leaves	Rice	[24]
Cytochalasin E (**162**)	$C_{28}H_{33}NO_7$	495.2257	*Xylaria* sp. XC-16	*T. sinensis*	Leaves	Rice	[24]
			Xylaria sp. XC-16	*T. sinensis*	Leaves	PD liquid medium	[42]
Chaetoglobosin C (**163**)	$C_{32}H_{36}N_2O_5$	528.2624	*B. dothidea* KJ-1	*M. azedarach L.*	Stem	Rice	[34]
Chaetoglobosin F (**164**)	$C_{32}H_{38}N_2O_5$	530.2781	*B. dothidea* KJ-1	*M. azedarach L.*	Stem	Rice	[34]
Phomopsichalasin D (**165**)	$C_{30}H_{37}NO_6$	509.2414	*Phomopsis* sp. xy22	*X. granatum*	Leaves	Rice	[59]
Phomopsichalasin E (**166**)	$C_{30}H_{37}NO_6$	507.2621	*Phomopsis* sp. xy22	*X. granatum*	Leaves	Rice	[59]
Phomopsichalasin F (**167**)	$C_{28}H_{37}NO_3$	435.2773	*Phomopsis* sp. xy21	*X. granatum*	Leaves	Rice	[59]
Phomopsichalasin G (**168**)	$C_{28}H_{37}NO_4$	451.2723	*Phomopsis* sp. xy21	*X. granatum*	Leaves	Rice	[59]
4′-hydroxy-deacetyl-18-deoxycytochalasin H (**169**)	$C_{28}H_{37}NO_4$	451.2723	*Phomopsis* sp. xy21	*X. granatum*	Leaves	Rice	[59]
deacetyl-18-deoxycytochalasin H (**170**)	$C_{28}H_{37}NO_3$	435.2773	*Phomopsis* sp. xy21	*X. granatum*	Leaves	Rice	[59]
18-deoxycytochalasin H (**171**)	$C_{30}H_{39}NO_4$	477.2879	*Phomopsis* sp. xy21	*X. granatum*	Leaves	Rice	[59]
cytochalasin H (**172**)	$C_{30}H_{39}NO_5$	493.2828	*Phomopsis* sp. xy22	*X. granatum*	Leaves	Rice	[59]
deacetylcytochalasin H (**173**)	$C_{28}H_{37}NO_4$	451.2723	*Phomopsis* sp. xy22	*X. granatum*	Leaves	Rice	[59]
epoxycytochalasin H (**174**)	$C_{30}H_{39}NO_5$	493.2828	*Phomopsis* sp. xy22	*X. granatum*	Leaves	Rice	[59]
Cytochalasin D (**175**)	$C_{30}H_{37}NO_6$	507.2621	*C. gloeosporioides*	*T. monadelpha*	Stem bark	Rice	[27]
Xylarisin B (**176**)	$C_{23}H_{35}NO_5$	405.2515	*Xylaria* sp. HNWSW-2	*X. granatum*	Stem	Rice	[41]
Epoxycytochalasin Z_{17} (**177**)	$C_{28}H_{33}NO_6$	479.2308	*Xylaria* sp. XC-16	*T. sinensis*	Leaves	PD liquid medium	[42]
Epoxycytochalasin Z_8 (**178**)	$C_{28}H_{35}NO_6$	481.2464	*Xylaria* sp. XC-16	*T. sinensis*	Leaves	PD liquid medium	[42]
Epoxyrosellichalasin (**179**)	$C_{28}H_{33}NO_6$	479.2308	*Xylaria* sp. XC-16	*T. sinensis*	Leaves	PD liquid medium	[42]
10-phenyl-[12]-cytochalasin Z_{16} (**180**)	$C_{28}H_{33}NO_5$	463.2359	*Xylaria* sp. XC-16	*T. sinensis*	Leaves	PD liquid medium	[42]
10-phenyl-[12]-cytochalasin Z_{17} (**181**)	$C_{28}H_{33}NO_5$	463.2359	*Xylaria* sp. XC-16	*T. sinensis*	Leaves	PD liquid medium	[42]
Cytochalasin K (**182**)	$C_{28}H_{33}NO_7$	495.2257	*Xylaria* sp. XC-16	*T. sinensis*	Leaves	PD liquid medium	[42]

**Aromatics**							
4,8-dihydroxy-1-tetralone (**183**)	$C_{10}H_{10}O_3$	178.0630	*A. fumigatus* LN-4	*M. azedarach*	Stem bark	PD liquid medium	[32]
trans-3,4-dihydro-3,4,8-trihydroxynaphtalen-1(2H)-one (**184**)	$C_{10}H_{10}O_4$	194.0579	*A. fumigatus* LN-4	*M. azedarach*	Stem bark	PD liquid medium	[32]
cis-3,4-dihydro-3,4,8-trihydroxynaphtalen-1(2H)-one (**185**)	$C_{10}H_{10}O_4$	194.0579	*A. fumigatus* LN-4	*M. azedarach*	Stem bark	PD liquid medium	[32]
Altenuene (**186**)	$C_{15}H_{16}O_6$	292.0947	*B. dothidea* KJ-1	*M. azedarach* L.	Stem	Rice	[34]
Altenusin (**187**)	$C_{15}H_{14}O_6$	290.0790	*B. dothidea* KJ-1	*M. azedarach* L.	Stem	Rice	[34]
Djalonensone (**188**)	$C_{14}H_{14}O_4$	246.0892	*B. dothidea* KJ-1	*M. azedarach* L.	Stem	Rice	[34]
Alternariol (**189**)	$C_{15}H_{12}O_5$	272.0685	*B. dothidea* KJ-1	*M. azedarach* L.	Stem	Rice	[34]
5′-methoxy-6-methylbiphenyl-3,4,3′-triol (**190**)	$C_{14}H_{10}O_5$	258.0528	*B. dothidea* KJ-1	*M. azedarach* L.	Stem	Rice	[34]
7-hydroxy-1-isochromanone 3587 (**191**)	$C_9H_{10}O_3$	166.0630	*B. dothidea* KJ-1	*M. azedarach* L.	Stem	Rice	[34]
5-(hydroxymethyl)-1H-pyrrole-2-carbaldehyde (**192**)	$C_6H_7NO_2$	125.0477	*B. dothidea* KJ-1	*M. azedarach* L.	Stem	Rice	[34]
5-hydroxymethylfurfural (**193**)	$C_6H_6O_3$	126.0317	*B. dothidea* KJ-1	*M. azedarach* L.	Stem	Rice	[34]
3-chloro-5-hydroxy-4-methoxyphenylacetic acid methyl ester (**194**)	$C_{10}H_{11}ClO_4$	230.0346	*Eupenicillium* sp. HJ002	*X. granatum*		Potato Liquid Medium (33g sea salt in 1L of potato infusion)	[22]
4-hydroxyphenylacetate (**195**)	$C_9H_{10}O_3$	166.0630	*Eupenicillium* sp. HJ002	*X. granatum*		Potato Liquid Medium (33g sea salt in 1L of potato infusion)	[22]
Cytosporone B (**196**)	$C_{18}H_{26}O_5$	322.1780	*Eupenicillium* sp. HJ002	*X. granatum*		Potato Liquid Medium (33g sea salt in 1L of potato infusion)	[22]
Eugenitol (**197**)	$C_{12}H_{12}O_4$	220.0736	*D. eucalyptorum* KY-9	*M. azedarach*	Leaves	Rice	[50]
Cytosporone C (**198**)	$C_{16}H_{22}O_2$	246.1620	*D. eucalyptorum* KY-9	*M. azedarach*	Leaves	Rice	[50]
4-hydroxyphenethyl alcohol (**199**)	$C_8H_{10}O_2$	138.0681	*D. eucalyptorum* KY-9	*M. azedarach*	Leaves	Rice	[50]

1-(4-hydroxyphenyl)ethane-1,2-diol (**200**)	$C_8H_{10}O_3$	154.0630	*D. eucalyptorum* KY-9	*M. azedarach*	Leaves	Rice	[50]
**Ester**							
(R)-butanedioic acid (**201**)	$C_{12}H_{20}O_4$	228.1362	Eupenicillium sp. HJ002	*X. granatum*		Potato Liquid Medium (33g sea salt in 1L of potato infusion)	[22]
**Quinols**							
Cytosporin D (**202**)	$C_{19}H_{30}O_5$	338.2093	*P. theae*	*T. longipes*	Leaves	Rice	[28]
Cytosporin F (**203**)	$C_{21}H_{32}O_6$	380.2199	*P. theae*	*T. longipes*	Leaves	Rice	[28]
Cytosporin G (**204**)	$C_{21}H_{32}O_7$	396.2148	*P. theae*	*T. longipes*	Leaves	Rice	[28]
Cytosporin H (**205**)	$C_{21}H_{32}O_7$	396.2148	*P. theae*	*T. longipes*	Leaves	Rice	[28]
Cytosporin I (**206**)	$C_{21}H_{32}O_7$	396.2148	*P. theae*	*T. longipes*	Leaves	Rice	[28]
Cytosporin J (**207**)	$C_{19}H_{30}O_6$	354.2042	*P. theae*	*T. longipes*	Leaves	Rice	[28]
Cytosporin K (**208**)	$C_{19}H_{30}O_6$	354.2042	*P. theae*	*T. longipes*	Leaves	Rice	[28]
**Alkaloids**							
Verruculogen (**209**)	$C_{27}H_{33}N_3O_7$	511.2319	*Penicillium* sp.	*M. azedarach*	Root bark	Rice	[46]
			A. fumigatus LN-4	*M. azedarach*	Stem bark	PD liquid medium	[32]
			P. brasilianum	*M. azaderach*	Root bark	Rice	[60]
Brasiliamide A (**210**)	$C_{24}H_{26}N_2O_6$	438.1791	*P. brasilianum*	*M. azedarach*	Root bark	Rice	[47]
Brasiliamide B (**211**)	$C_{22}H_{22}N_2O_6$	410.1478	*P. brasilianum*	*M. azedarach*	Root bark	Rice	[47]
Fumitremorgin C (**212**)	$C_{22}H_{25}N_3O_3$	379.1896	*A. fumigatus* LN-4	*M. azedarach*	Stem bark	PD liquid medium	[32]
Cyclotryprostatin A (**213**)	$C_{22}H_{25}N_3O_5$	411.1794	*A. fumigatus* LN-4	*M. azedarach*	Stem bark	PD liquid medium	[32]
Cyclotryprostatin B (**214**)	$C_{23}H_{27}N_3O_5$	425.1951	*A. fumigatus* LN-4	*M. azedarach*	Stem bark	PD liquid medium	[32]
Verrulocogen TR-2 (**215**)	$C_{22}H_{27}N_3O_6$	429.1900	*A. fumigatus* LN-4	*M. azedarach*	Stem bark	PD liquid medium	[32]
12β-hydroxyverruculogen TR-2 (**216**)	$C_{22}H_{27}N_3O_6$	429.1900	*A. fumigatus* LN-4	*M. azedarach*	Stem bark	PD liquid medium	[32]
12β-hydroxy-13α-methoxyverruculogen TR-2 (**217**)	$C_{23}H_{29}N_3O_6$	443.2056	*A. fumigatus* LN-4	*M. azedarach*	Stem bark	PD liquid medium	[32]
Fumitremorgin B (**218**)	$C_{27}H_{33}N_3O_5$	479.2420	*A. fumigatus* LN-4	*M. azedarach*	Stem bark	PD liquid medium	[32]
Tryprostatin A (**219**)	$C_{22}H_{27}N_3O_3$	381.2052	*A. fumigatus* LN-4	*M. azedarach*	Stem bark	PD liquid medium	[32]
Cyclo-L-tryptophyl-L-proline (**220**)	$C_{16}H_{17}N_3O_2$	283.1321	*A. fumigatus* LN-4	*M. azedarach*	Stem bark	PD liquid medium	[32]
Terezine D (**221**)	$C_{19}H_{23}N_3O_2$	325.1790	*A. fumigatus* LN-4	*M. azedarach*	Stem bark	PD liquid medium	[32]
Fumiquinazoline F (**222**)	$C_{21}H_{18}N_4O_2$	358.1430	*A. fumigatus* LN-4	*M. azedarach*	Stem bark	PD liquid medium	[32]
Fumiquinazoline G (**223**)	$C_{21}H_{18}N_4O_2$	358.1430	*A. fumigatus* LN-4	*M. azedarach*	Stem bark	PD liquid medium	[32]

Fumiquinazoline D (**224**)	$C_{24}H_{21}N_5O_4$	443.1594	*A. fumigatus* LN-4	*M. azedarach*	Stem bark	PD liquid medium	[32]
Fumiquinazoline A (**225**)	$C_{24}H_{23}N_5O_4$	445.1750	*A. fumigatus* LN-4	*M. azedarach*	Stem bark	PD liquid medium	[32]
3-hydroxyfumiquinazoline A (**226**)	$C_{24}H_{23}N_5O_5$	461.1699	*A. fumigatus* LN-4	*M. azedarach*	Stem bark	PD liquid medium	[32]
6-methoxyspirotryprostatin B (**227**)	$C_{22}H_{23}N_3O_4$	393.1689	*A. fumigatus* LN-4	*M. azedarach*	Stem bark	PD liquid medium	[32]
Spiro [5H,10H-dipyrrolo[1,2-α:1′,2′-d]pyrazine-2-(3H),2′-[2H]indole]-3′,5,10(1′H)-trione (**228**)	$C_{22}H_{25}N_3O_6$	427.1743	*A. fumigatus* LN-4	*M. azedarach*	Stem bark	PD liquid medium	[32]
Pseurotin A (**229**)	$C_{22}H_{25}NO_8$	431.1580	*A. fumigatus* LN-4	*M. azedarach*	Stem bark	PD liquid medium	[32] [32]
Pseurotin A1 (**230**)	$C_{22}H_{25}NO_8$	431.1580	*A. fumigatus* LN-4	*M. azedarach*	Stem bark	PD liquid medium	[32]
Tryptoquivaline O (**231**)	$C_{22}H_{18}N_4O_4$	402.1328	*A. fumigatus* LN-4	*M. azedarach*	Stem bark	PD liquid medium	[32]
Fumifaclavine B (**232**)	$C_{16}H_{20}N_2O$	256.1576	*A. fumigatus* LN-4	*M. azedarach*	Stem bark	PD liquid medium	[32]
Bisdethiobis(methylthio)gliotoxin (**233**)	$C_{15}H_{20}N_2O_4S_2$	356.0864	*A. fumigatus* LN-4	*M. azedarach*	Stem bark	PD liquid medium	[32]
Cyclo-(Pro-Gly) (**234**)	$C_7H_{10}N_2O_2$	154.0742	*A. fumigatus* LN-4	*M. azedarach*	Stem bark	PD liquid medium	[32]
Cyclo-(Pro-Ala) (**235**)	$C_8H_{12}N_2O_2$	168.0899	*A. fumigatus* LN-4	*M. azedarach*	Stem bark	PD liquid medium	[32]
Cyclo-(D-Pro-L-Ala) (**236**)	$C_8H_{12}N_2O_2$	168.0899	*A. fumigatus* LN-4	*M. azedarach*	Stem bark	PD liquid medium	[32]
Cyclo-(Pro-Ser) (**237**)	$C_9H_{14}N_2O_3$	198.1004	*A. fumigatus* LN-4	*M. azedarach*	Stem bark	PD liquid medium	[32]
Cyclo-(Ser-trans-4-OH-Pro) (**238**)	$C_9H_{14}N_2O_4$	214.0954	*A. fumigatus* LN-4	*M. azedarach*	Stem bark	PD liquid medium	[32]
Cyclo-(Leu-4-OH-Pro) (**239**)	$C_{12}H_{20}N_2O_3$	240.1474	*A. fumigatus* LN-4	*M. azedarach*	Stem bark	PD liquid medium	[32]
Cyclo-(Ala-trans-4-OH-Pro) (**240**)	$C_8H_{12}N_2O_3$	184.0848	*A. fumigatus* LN-4	*M. azedarach*	Stem bark	PD liquid medium	[32]
Cyclo-(cis-OH-D-Pro-L-Phe) (**241**)	$C_{14}H_{16}N_2O_3$	260.1161	*A. fumigatus* LN-4	*M. azedarach*	Stem bark	PD liquid medium	[32]
Cyclo-(Gly-Phe) (**242**)	$C_{11}H_{12}N_2O_2$	204.0899	*A. fumigatus* LN-4	*M. azedarach*	Stem bark	PD liquid medium	[32]
Cyclo-(Pro-tans-4-OH-Pro) (**243**)	$C_{10}H_{14}N_2O_3$	210.1004	*A. fumigatus* LN-4	*M. azedarach*	Stem bark	PD liquid medium	[32]
Cyclo-(Gly-Ala) (**244**)	$C_5H_8N_2O_2$	128.0586	*A. fumigatus* LN-4	*M. azedarach*	Stem bark	PD liquid medium	[32]
Uracil (**245**)	$C_4H_4N_2O_2$	410.1478	*A. fumigatus* LN-4	*M. azedarach*	Stem bark	PD liquid medium	[32]
Rohitukine (**246**)	$C_{16}H_{19}NO_5$	305.1263	*F. proliferatum* MTCC 9690	*D. binectariferum Hook.f*	Bark	PDB	[61]
			F. oxysporum MTCC 11383	*D. binectariferum Hook.f*	Leaves	Broth	[61]
			*F. solani*MTCC 11385	*D. binectariferum Hook.f*	Fruit	Broth	[61]
			F. oxysporum MTCC 11384	*D. binectariferum Hook.f*	Bark	Broth	[61]

			G. fujikurai MTCC 11382	*Amoora rohituka*	Bark	Broth	[61]
Rohitukine *N*-oxide (**247**)	$C_{16}H_{19}NO_5$	321.1212	*F. oxysporum* MTCC 11383	*D. binectariferum Hook.f*	Leaves	Broth	[61]
			*F. solani*MTCC 11385	*D. binectariferum Hook.f*	Fruit	Broth	[61]
			F. oxysporum MTCC 11384	*D. binectariferum Hook.f*	Bark	Broth	[61]
			G. fujikurai MTCC 11382	*Amoora rohituka*	Bark	Broth	[61]
Flavopiridol (**248**)	$C_{21}H_{20}ClNO_5$	401.1030	*F. oxysporum* MTCC 11383	*D. binectariferum Hook.f*	Leaves	Broth	[61]
			F. solani MTCC 11385	*D. binectariferum Hook.f*	Fruit	Broth	[61]
			F. oxysporum MTCC 11384	*D. binectariferum Hook.f*	Bark	Broth	[61]
			G. fujikurai MTCC 11382	*A. rohituka*	Bark	Broth	[61]
2-(furan-2-yl)-6-(2*S*,3*S*,4-trihydroxybutyl)pyrazine (**249**)	$C_{12}H_{14}N_2O_4$	250.0954	*J. endophytica* 161111	*X. granatum*	Root	Broth (glucose 2%, yeast extract 0.5%, peptone 0.5%, KNO_3 1.5%, $CaCO_3$ 0.4%, and NaCl 0.4% (pH 7.2))	[62]
2-(furan-2-yl)-5-(2*S*,3*S*,4-trihydroxybutyl)pyrazine (**250**)	$C_{12}H_{14}N_2O_4$	250.0954	*J. endophytica* 161111	*X. granatum*	Root	Broth (glucose 2%, yeast extract 0.5%, peptone 0.5%, KNO_3 1.5%, $CaCO_3$ 0.4%, and NaCl 0.4% (pH 7.2))	[62]

(*S*)-4-isobutyl-3-oxo-3,4-dihydro-1 H-pyrrolo[2,1-c][1,4]oxazine-6-carbaldehyde (**251**)	$C_{13}H_{17}NO_3$	235.1208	*J. endophytica* 161111	*X. granatum*	Root	Broth (glucose 2%, yeast extract 0.5%, peptone 0.5%, KNO_3 1.5%, $CaCO_3$ 0.4%, and NaCl 0.4% (pH 7.2))	[62]
(*S*)-4-isopropyl-3-oxo-3,4-dihydro-1H-pyrrolo [2,1-c][1,4]oxazine-6-carbaldehyde (**252**)	$C_{12}H_{15}NO_3$	221.1052	*J. endophytica* 161111	*X. granatum*	Root	Broth (glucose 2%, yeast extract 0.5%, peptone 0.5%, KNO_3 1.5%, $CaCO_3$ 0.4%, and NaCl 0.4% (pH 7.2))	[62]
(4*S*)-4-(2-methylbutyl)-3-oxo-3,4-dihydro-1H-pyrrolo [2,1-c][1,4]oxazine-6-carbaldehyde (**253**)	$C_{13}H_{17}NO_3$	235.1208	*J. endophytica* 161111	*X. granatum*	Root	Broth (glucose 2%, yeast extract 0.5%, peptone 0.5%, KNO_3 1.5%, $CaCO_3$ 0.4%, and NaCl 0.4% (pH 7.2))	[62]
(*S*)-4-benzyl-3-oxo-3,4-dihydro-1H-pyrrolo[2,1-c] [1,4]oxazine-6-carbaldehyde (**254**)	$C_{16}H_{15}NO_3$	269.1052	*J. endophytica* 161111	*X. granatum*	Root	Broth (glucose 2%, yeast extract 0.5%, peptone 0.5%, KNO_3 1.5%, $CaCO_3$ 0.4%, and NaCl 0.4% (pH 7.2))	[62]
Flazin (255)	$C_{18}H_{14}N_2O_4$	322.0954	*J. endophytica* 161111	*X. granatum*	Root	Broth (glucose 2%, yeast extract 0.5%, peptone 0.5%, KNO_3 1.5%, $CaCO_3$ 0.4%, and NaCl 0.4% (pH 7.2))	[62]
Perlolyrine (**256**)	$C_{17}H_{14}N_2O_2$	278.1055	*J. endophytica* 161111	*X. granatum*	Root	Broth (glucose 2%, yeast extract 0.5%, peptone 0.5%, KNO_3 1.5%, $CaCO_3$ 0.4%, and NaCl 0.4% (pH 7.2))	[62]
1-hydroxy-β-carboline (**257**)	$C_{11}H_8N_2O$	184.0637	*J. endophytica* 161111	*X. granatum*	Root	Broth (glucose 2%, yeast extract 0.5%, peptone 0.5%, KNO_3 1.5%, $CaCO_3$ 0.4%, and NaCl 0.4% (pH 7.2))	[62]

Lumichrome (**258**)	$C_{12}H_{10}N_4O_2$	242.0804	*J. endophytica* 161111	*X. granatum*	Root	Broth (glucose 2%, yeast extract 0.5%, peptone 0.5%, KNO_3 1.5%, $CaCO_3$ 0.4%, and NaCl 0.4% (pH 7.2))	[62]
1H-indole-3-carboxaldehyde (**259**)	C_9H_7NO	145.0528	*J. endophytica* 161111	*X. granatum*	Root	Broth (glucose 2%, yeast extract 0.5%, peptone 0.5%, KNO_3 1.5%, $CaCO_3$ 0.4%, and NaCl 0.4% (pH 7.2))	[62]
[14,15], 2-hydroxy-1-(1H-indol-3-yl) ethenone (**260**)	$C_{10}H_9NO_2$	175.0633	*J. endophytica* 161111	*X. granatum*	Root	Broth (glucose 2%, yeast extract 0.5%, peptone 0.5%, KNO_3 1.5%, $CaCO_3$ 0.4%, and NaCl 0.4% (pH 7.2))	[62]
5-(methoxymethyl)-1H-pyrrole-2-carbaldehyde (**261**)	$C_8H_{11}NO$	137.0841	*J. endophytica* 161111	*X. granatum*	Root	Broth (glucose 2%, yeast extract 0.5%, peptone 0.5%, KNO_3 1.5%, $CaCO_3$ 0.4%, and NaCl 0.4% (pH 7.2))	[62]
Asperazine (**262**)	$C_{40}H_{36}N_6O_4$	664.2798	*Aspergillus* sp. KJ-9	*M. azedarach*	Stem Bark		[44]
(*R*)-3-hydroxybutanonitrile (**263**)	C_4H_7NO	85.0528	*Aspergillus* sp. KJ-9	*M. azedarach*	Stem Bark		[34]
3-hydroxy-2-methoxy-5-methylpyridin-2(1H)-one (**264**)	$C_7H_9NO_3$	155.0582	*B. dothidea* KJ-1	*M. azedarach L.*	Stem	Rice	[34]
3-hydroxy-*N*-(1-hydroxy-3-methylpentan-2-yl)-5-oxohexanamide (**265**)	$C_{12}H_{23}NO_4$	245.1627	*B. dothidea* KJ-1	*M. azedarach L.*	Stem	Rice	[34]
3-hydroxy-N-(1-hydroxy-4-methylpentan-2-yl)-5-oxohexanamide (**266**)	$C_{12}H_{23}NO_4$	245.1627	*B. dothidea* KJ-1	*M. azedarach L.*	Stem	Rice	[34]
(2*S*,3a*R*,6*S*,7a*S*) -6-acetamido-octahydro-1,3-benzothiazol-2-yl 2-(adamantan-1-yl) acetamide (**267**)	$C_{21}H_{33}N_3O_2S$	391.2293	*Emericella* sp.	*A. indica A. Juss*	Twig	Liquid media containing 10 g glucose, 5 g peptone, 3 g malt extract, and 3 g yeast extract	[63]
**Nitro Compounds**							
3-nitropropionic acid (**268**)	$C_3H_5NO_4$	119.0219	*Phomopsis longicolla* FJ2759	*T. elegans* A. Juss ssp. elegans	Leaves	BD broth	[28]

**Fatty Acid & Sugars**							
Fusaroside (**269**)	$C_{34}H_{54}O_{13}$	670.3564	*Fusarium* sp. LN-11	*M. azedarach*	Leaves	Liquid Culture	[21]
Phalluside (**270**)	$C_{44}H_{81}NO_8$	751.5962	*Fusarium* sp. LN-11	*M. azedarach*	Leaves	Liquid Culture	[21]
(9*R*,10*R*,7*E*)-6,9,10-trihydroxyoctadec-7-enoic acid (**271**)	$C_{18}H_{34}O_5$	330.2406	*Fusarium* sp. LN-11	*M. azedarach*	Leaves	Liquid Culture	[21]
Porrigenic acid (**272**)	$C_{18}H_{30}O_4$	310.2144	*Fusarium* sp. LN-11	*M. azedarach*	Leaves	Liquid Culture	[21]
(9*Z*)-2,3-dihydroxypropyl octadeca-9-enoate (**273**)	$C_{21}H_{40}O_4$	356.2927	*Fusarium* sp. LN-11	*M. azedarach*	Leaves	Liquid Culture	[21]
Cerebroside C (**274**)	$C_{39}H_{71}NO_9$	697.5129	*B. dothidea* KJ-1	*M. azedarach L.*	Stem	Rice	[44]
Eucalyptacid A (**275**)	$C_{17}H_{32}O_5$	316.2250	*D. eucalyptorum* KY-9	*M. azedarach*	Leaves	Rice	[50]
Phomopene (**276**)	$C_{10}H_{18}O_3$	186.1256	*D. eucalyptorum* KY-9	*M. azedarach*	Leaves	Rice	[50]

Table S2. Antimicrobial Activity of Compounds that have been evaluated against several fungi and bacteria.

Compounds	Antimicrobial	Ref.
Helvolic acid (**4**)	Antifungal activity (MIC: μg/mL)<br>*B. cinerea* (6.25), *A. solani* (12.5), *A. alternata* (6.25), *C. gloeosporioides* (6.25), *F. solani*(50), *F. oxysporum* f. sp. *niveum* (12.5), *F. oxysporum* f. sp. *vasinfectum* (25), *G. saubinettii* (6.25); Positive control (MIC: μg/mL) carbendazim: 12.5, 12.5, 6.25, 6.25, 25, 12.5, 25, 6.25, respectively hymexazol: 12.5, 12.5, 12.5, 12.5, 50, 12.5, 2.5, 12.5, respectively	[32]
Pycnophorin (**7**)	Antifungal activity (MIC: μM)<br>*B. cinerea* (100), *A. solani* (6.25), *C. gloeosporioides* (200), *G. saubinettii* (200); Positive control (MIC: μM) carbendazim: 12.5, 1.57, 1.57, 6.25, respectively. hymexazol: 200, 6.25, >200, >200, respectively. toosendanin: 200, 6.25, 200, 200, respectively<br><br>Antibacterial activity (MIC: μM)<br>*E. coli* (200), *B. subtilis* (25), *S. aureus* (25), *B. cereus* (50); Positive control (MIC: μM) ampicillin: 1.57, 1.57, 1.57, 12.5, respectively streptomycin sulfate: 1.57, 1.57, 1.57, 1.57, respectively	[34]
(1*S*,4*S*,5*R*,7*R*,10*R*,11*R*)-guaiane-5,10,11,12-tetraol **(8)**	Antifungal activity (MIC: μg/mL)<br>*C. albicans* YM 2005 (256), *A. niger* YM 3029 (128), *P. oryzae* YM 3051 (256), *F. avenaceum* YM 3065 (512), *H. compactum* YM 3077 (128); Positive control (MIC: μg/mL) nystatin: 8, 8, 8, 16, 8, respectively	[35]
(1*S*,4*S*,5*S*,7*R*,10*R*,11*S*)-guaiane-1,10,11,12-tetraol (**9**)	Antifungal activity (MIC: μg/mL)<br>*C. albicans* YM 2005 (256), *A. niger* YM 3029 (128), *P. oryzae* YM 3051 (256), *F. avenaceum* YM 3065 (512),<br>*H. compactum* YM 3077 (128) :<br>Positive control (MIC: μg/mL) nystatin: 8, 8, 8, 16, 8, respectively	[35]
(1*S*,4*S*,5*R*,7*R*,10*R*,11*S*)-guaiane-5,10,11,12-tetraol (**10**)	Antifungal activity (MIC: μg/mL)<br>*C. albicans* YM 2005 (32), *A. niger* YM 3029 (64), *P. oryzae* YM 3051 (256), *F. avenaceum* YM 3065 (>512), *H. compactum* YM 3077 (64); Positive control (MIC: μg/mL) nystatin: 8, 8, 8, 16, 8, respectively	[35]
(1*S*,4*S*,5*S*,7*R*,10*R*,11*R*)-guaiane-1,10,11,12-tetraol (**11**)	Antifungal activity (MIC: μg/mL)<br>*C. albicans* YM 2005 (128), *A. niger* YM 3029 (256),<br>*P. oryzae* YM 3051 (128), *F. avenaceum* YM 3065 (512),<br>*H. compactum* YM 3077 (256);<br>Positive control (MIC: μg/mL) nystatin: 8, 8, 8, 16, 8, respectively	[35]
(1*R*,3*S*,4*R*,5*S*,7*R*,10*R*,11*S*)-guaiane-3,10,11,12-tetraol (**12**)	Antifungal activity (MIC: μg/mL)<br>*C. albicans* YM 2005 (64), *A. niger* YM 3029 (64), *P. oryzae* YM 3051 (256), *F. avenaceum* YM 3065 (>512), *H. compactum* YM 3077 (256); Positive control (MIC: μg/mL) nystatin: 8, 8, 8, 16, 8, respectively	[35]
(1*R*,3*R*,4*R*,5*S*,7*R*,10*R*,11*R*)-guaiane-3,10,11,12-tetraol (**13**)	Antifungal activity (MIC: μg/mL)<br>*C. albicans* YM 2005 (64), *A. niger* YM 3029 (512),<br>*P. oryzae* YM 3051 (256), *F. avenaceum* YM 3065 (>512),<br>*H. compactum* YM 3077 (128)<br>Positive control (MIC: μg/mL) nystatin: 8, 8, 8, 16, 8, respectively	[35]
(1*R*,4*S*,5*S*,7*S*,9*R*,10*S*,11*R*)-guaiane-9,10,11,12-tetraol (**14**)	Antifungal activity (MIC: μg/mL)<br>*C. albicans* YM 2005 (128), *A. niger* YM 3029 (512),<br>*P. oryzae* YM 3051 (128), *F. avenaceum* YM 3065 (512),	[35]

	H. compactum YM 3077 (128);<br>Positive control (MIC: µg/mL) nystatin: 8, 8, 8, 16, 8, respectively	
(1*R*,4*S*,5*S*,7*R*,10*R*,11*S*)-guaiane-10,11,12-triol (**15**)	Antifungal activity (MIC: µg/mL)<br>*C. albicans* YM 2005 (32), *A. niger* YM 3029 (128),<br>*P. oryzae* YM 3051 (512), *F. avenaceum* YM 3065 (>512), *H. compactum* YM 3077 (256);<br>Positive control (MIC: µg/mL) nystatin: 8, 8, 8, 16, 8, respectively	[35]
(1*R*,4*S*,5*S*,7*R*,10*R*,11*R*)-guaiane-10,11,12-triol (**16**)	Antifungal activity (MIC: µg/mL)<br>*C. albicans* YM 2005 (128), *A. niger* YM 3029 (256),<br>*P. oryzae* YM 3051 (512), *F. avenaceum* YM 3065 (>512),<br>*H. compactum* YM 3077 (128);<br>Positive control (MIC: µg/mL) nystatin: 8, 8, 8, 16, 8, respectively	[35]
14*α*,16-epoxy-18-norisopimar-7-en-4*α*-ol (**17**)	Antifungal activity (MIC: µg/mL)<br>*C. albicans* YM 2005 (128), *A. niger* YM 3029 (>512),<br>*P. oryzae* YM 3051 (256), *F. avenaceum* YM 3065 (512),<br>*H. compactum* YM 3077 (256);<br>Positive control (MIC: µg/mL) nystatin: 8, 8, 8, 16, 8, respectively	[35]
16-O-sulfo-18-norisopimar-7-en-4*α*,16-diol (**18**)	Antifungal activity (MIC: µg/mL)<br>*C. albicans* YM 2005 (64), *A. niger* YM 3029 (64),<br>*P. oryzae* YM 3051 (256), *F. avenaceum* YM 3065 (64), *H. compactum* YM 3077 (128);<br>Positive control (MIC: µg/mL) nystatin: 8, 8, 8, 16, 8, respectively	[35]
9-deoxy-hymatoxin A (**19**)	Antifungal activity (MIC: µg/mL)<br>*C. albicans* YM 2005 (64), *A. niger* YM 3029 (128),<br>*P. oryzae* YM 3051 (32), *F. avenaceum* YM 3065 (128),<br>*H. compactum* YM 3077 (64);<br>Positive control (MIC: µg/mL) nystatin: 8, 8, 8, 16, 8, respectively	[35]
guaiane-2,10,11,12-tetraol (**20**)	Antifungal activity (MIC: µg/mL)<br>*C. albicans* YM 2005 (16),*A. niger* YM 3029 (32),<br>*P. oryzae* YM 3051 (16), *F. avenaceum* YM 3065 (64),<br>*H. compactum* YM 3077 (64);<br>Positive control (MIC: µg/mL) nystatin: 8, 8, 8, 16, 8, respectively	[36]
guaiane-2,4,10,11,12-pentaol (**21**)	Antifungal activity (MIC: µg/mL)<br>*C. albicans* YM 2005 (512), *A. niger* YM 3029 (256),<br>*P. oryzae* YM 3051 (128), *F. avenaceum* YM 3065 (>512),<br>*H. compactum* YM 3077 (256);<br>Positive control (MIC: µg/mL) nystatin: 8, 8, 8, 16, 8, respectively	[36]
guaiane-4,5,10,11,12-pentaol **(22**)	Antifungal activity (MIC: µg/mL)<br>*C. albicans* YM 2005 (128), *A. niger* YM 3029 (>512),<br>*P. oryzae* YM 3051 (256), *F. avenaceum* YM 3065 (>512), *H. compactum* YM 3077 (256);<br>Positive control (MIC: µg/mL) nystatin: 8, 8, 8, 16, 8, respectively	[36]
guaiane-1,5,10,11,12-pentaol (**23**)	Antifungal activity (MIC: µg/mL)<br>*C. albicans* YM 2005 (64), *A. niger* YM 3029 (64),<br>*P. oryzae* YM 3051 (256), *F. avenaceum* YM 3065 (>512),<br>*H. compactum* YM 3077 (64);	[36]

	Positive control (MIC: μg/mL) nystatin: 8, 8, 8, 16, 8, respectively	
11-methoxyguaiane-4,10,12-triol (**24**)	Antifungal activity (MIC: μg/mL)<br>*C. albicans* YM 2005 (32), *A. niger* YM 3029 (256),<br>*P. oryzae* YM 3051 (32), *F. avenaceum* YM 3065 (>512),<br>*H. compactum* YM 3077 (64)<br>Positive control (MIC: μg/mL) nystatin: 8, 8, 8, 16, 8, respectively	[36]
(7*R*,10*S*)-7,10-epoxysydonic acid (**47**)	Antibacterial activity against *S. aureus* ATCC 25923 with IC_{50} value of >50 μM; Positive control (IC_{50}: μM) penicillin: 23.6,rocephin: 1.3	[40]
(7*S*,10*S*)-7,10-epoxysydonic acid (**48**)	Antibacterial activity against *S. aureus* ATCC 25923 with IC_{50} value of 32.2 μM; Positive control (IC_{50}: μM) penicillin: 23.6, rocephin: 1.3	[40]
(7*R*,11*S*)-7,12-epoxysydonic acid (**49**)	Antibacterial activity against *S. aureus* ATCC 25923 with IC_{50} value of 36.0 μM; Positive control (IC_{50}: μM), penicillin: 23.6, rocephin: 1.3	[40]
(7*S*,11*S*)-7,12-epoxysydonic acid (**50**)	Antibacterial activity against *S. aureus* ATCC 25923 with IC_{50} value of >50 μM; Positive control (IC_{50}: μM), penicillin: 23.6, rocephin: 1.3	[40]
7-deoxy-7,14-didehydro-12-hydroxysydonic acid (**51**)	Antibacterial activity against *S. aureus* ATCC 25923 with IC_{50} value of 41.9 μM<br>Positive control (IC_{50}: μM) penicillin: 23.6, rocephin: 1.3	[40]
(*Z*)-7-deoxy-7,8-didehydro-12-hydro-xysydonic acid (**52**)	Antibacterial activity against *S. aureus* ATCC 25923 with IC_{50} value of >50 μM; Positive control (IC_{50}: μM) penicillin: 23.6, rocephin: 1.3	[40]
(*E*)-7-deoxy-7,8-didehydro-12-hydroxysydonic acid (**53**)	Antibacterial activity against *S. aureus* ATCC 25923 with IC_{50} value of 31.5 μM;<br>Positive control (IC_{50}: μM) penicillin: 23.6, rocephin: 1.3	[40]
(+)-1-hydroxyboivinianic acid (**54**)	Antibacterial activity against *S. aureus* ATCC 25923 with IC_{50} value of >50 μM; Positive control (IC_{50}: μM) penicillin: 23.6, rocephin: 1.3	[40]
engyodontiumone I (**55**)	Antibacterial activity against *S. aureus* ATCC 25923 with IC_{50} value of 33.4 μM; Positive control (IC_{50}: μM) penicillin: 23.6, rocephin: 1.3	[40]
(+)-sydonic acid (**56**)	Antibacterial activity against *S. aureus* ATCC 25923 with IC_{50} value of >50 μM; Positive control (IC_{50}: μM) penicillin: 23.6, rocephin: 1.3	[40]
(+)-hydroxysydonic acid (**57**)	Antibacterial activity against *S. aureus* ATCC 25923 with IC_{50} value of 34.0 μM, Positive control (IC_{50}: μM) penicillin: 23.6, rocephin: 1.3	[40]
(−)-(7S)-10-hydroxysydonic acid (**58**)	Antibacterial activity against *S. aureus* ATCC 25923 with IC_{50} value of 36.3 μM; Positive control (IC_{50}: μM) penicillin: 23.6; rocephin: 1.3	[40]
Verruculogen (**209**)	Antibacterial activity<br>Bacteriostatic effect for *E. coli* at a dosage of 250 μg/mL.<br>Control: penicillin, vancomycin, and tetracycline tested at a conc. of 25 μg/Ml<br>Antifungal activity (MIC: μg/mL)<br>*B. cinerea* (6.25), *A. solani* (12.5), *A. alternata* (6.25), *C. gloeosporioides* (6.25), *F. solani* (50), *F. oxysporum* f. sp. *niveum* (12.5), *F. oxysporum* f. sp. *vasinfectum* (25), *G. saubinettii* (6.25)<br>Positive control (MIC: μg/mL)	[46]

	carbendazim: 12.5, 12.5, 6.25, 6.25, 25, 12.5, 25, 6.25, respectively<br>hymexazol: 12.5, 12.5, 12.5, 12.5, 50, 12.5, 2.5, 12.5, respectively	
Fumitremorgin C (**212**)	Antifungal activity (MIC: μg/mL)<br>*B. cinerea* (12.5), *A. solani* (25), *A. alternata* (12.5), *C. gloeosporioides* (12.5), *F. solani*(50), *F. oxysporum* f. sp. *niveum* (25), *F. oxysporum* f. sp. *vasinfectum* (50), *G. saubinettii* (12.5)<br>Positive control (MIC: μg/mL)<br>carbendazim: 12.5, 12.5, 6.25, 6.25, 25, 12.5, 25, 6.25, respectively<br>hymexazol: 12.5, 12.5, 12.5, 12.5, 50, 12.5, 2.5, 12.5, respectively	[32]
Cyclotryprostatin A (**213**)	Antifungal activity (MIC: μg/mL)<br>*B. cinerea* (50), *A. solani* (50), *A. alternata* (25), *C. gloeosporioides* (50), *F. solani*(>100),<br>*F. oxysporum* f. sp. *niveum* (50), *F. oxysporum* f. sp. *vasinfectum* (100), *G. saubinettii* (50)<br>Positive control (MIC: μg/mL)<br>carbendazim: 12.5, 12.5, 6.25, 6.25, 25, 12.5, 25, 6.25, respectively<br>hymexazol: 12.5, 12.5, 12.5, 12.5, 50, 12.5, 2.5, 12.5, respectively	[32]
Cyclotryprostatin B (**214**)	Antifungal activity (MIC: μg/mL)<br>*B. cinerea* (12.5), *A. solani* (12.5), *A. alternata* (12.5), *C. gloeosporioides* (12.5), *F. solani*(25), *F. oxysporum* f. sp. *niveum* (12.5), *F. oxysporum* f. sp. *vasinfectum* (25), *G. saubinettii* (12.5)<br>Positive control (MIC: μg/mL)<br>carbendazim: 12.5, 12.5, 6.25, 6.25, 25, 12.5, 25, 6.25, respectively<br>hymexazol: 12.5, 12.5, 12.5, 12.5, 50, 12.5, 2.5, 12.5, respectively	[32]
Verrulocogen TR-2 (**215**)	Antifungal activity (MIC: μg/mL)<br>*B. cinerea* (12.5), *A. solani* (12.5), *A. alternata* (6.25), *C. gloeosporioides* (12.5), *F. solani*(25), *F. oxysporum* f. sp. *niveum* (12.5), *F. oxysporum* f. sp. *vasinfectum* (25), *G. saubinettii* (12.5)<br>Positive control (MIC: μg/mL)<br>carbendazim: 12.5, 12.5, 6.25, 6.25, 25, 12.5, 25, 6.25, respectively<br>hymexazol: 12.5, 12.5, 12.5, 12.5, 50, 12.5, 2.5, 12.5, respectively	[32]
12β-hydroxyverruculogen TR-2 (**216**)	Antifungal activity (MIC: μg/mL)<br>*B. cinerea* (25), *A. solani* (25), *A. alternata* (25), *C. gloeosporioides* (25), *F. solani*(50), *F. oxysporum* f. sp. *niveum* (25), *F. oxysporum* f. sp. *vasinfectum* (25), *G. saubinettii* (25)<br>Positive control (MIC: μg/mL)<br>carbendazim: 12.5, 12.5, 6.25, 6.25, 25, 12.5, 25, 6.25, respectively<br>hymexazol: 12.5, 12.5, 12.5, 12.5, 50, 12.5, 2.5, 12.5, respectively	[32]
12β-hydroxy-13α-methoxyverruculogen TR-2 (**217**)	Antifungal activity (MIC: μg/mL)<br>*B. cinerea* (6.25), *A. solani* (6.25), *A. alternata* (6.25), *C. gloeosporioides* (6.25),<br>*F. solani*(25), *F. oxysporum* f. sp. *niveum* (12.5), *F. oxysporum* f. sp. *vasinfectum* (25), *G. saubinettii* (6.25)<br>Positive control (MIC: μg/mL)<br>carbendazim: 12.5, 12.5, 6.25, 6.25, 25, 12.5, 25, 6.25, respectively<br>hymexazol: 12.5, 12.5, 12.5, 12.5, 50, 12.5, 2.5, 12.5, respectively	[32]
Fumitremorgin B (**218**)	Antifungal activity (MIC: μg/mL)<br>*B. cinerea* (6.25), *A. solani* (6.25), *A. alternata* (6.25), *C. gloeosporioides* (12.5), *F. solani*(100), *F. oxysporum* f. sp. *niveum* (25), *F. oxysporum* f. sp. *vasinfectum* (50), *G. saubinettii* (12.5)	[32]

	Positive control (MIC: µg/mL)<br>carbendazim: 12.5, 12.5, 6.25, 6.25, 25, 12.5, 25, 6.25, respectively<br>hymexazol: 12.5, 12.5, 12.5, 12.5, 50, 12.5, 2.5, 12.5, respectively	
Tryprostatin A (**219**)	Antifungal activity (MIC: µg/mL)<br>*B. cinerea*, *A. solani*, *A. alternata* , *C. gloeosporioides*, *Fusarium solani*, *F. oxysporum* f. sp. *niveum*, *F. oxysporum* f. sp. *vasinfectum*, *G. saubinettii* (>100)<br>Positive control (MIC: µg/mL)<br>carbendazim: 12.5, 12.5, 6.25, 6.25, 25, 12.5, 25, 6.25, respectively<br>hymexazol: 12.5, 12.5, 12.5, 12.5, 50, 12.5, 2.5, 12.5, respectively	[32]
Cyclo-L-tryptophyl-L-proline (**220**)	Antifungal activity (MIC: µg/mL)<br>*B. cinerea*, *A. solani*, *A. alternata* , *C. gloeosporioides*, *Fusarium solani*, *F. oxysporum* f. sp. *niveum*, *F. oxysporum* f. sp. *vasinfectum*, *G. saubinettii* (>100)<br>Positive control (MIC: µg/mL)<br>carbendazim: 12.5, 12.5, 6.25, 6.25, 25, 12.5, 25, 6.25, respectively<br>hymexazol: 12.5, 12.5, 12.5, 12.5, 50, 12.5, 2.5, 12.5, respectively	[32]
Terezine D (**221**)	Antifungal activity (MIC: µg/mL)<br>*B. cinerea*, *A. solani*, *A. alternata* , *C. gloeosporioides*, *Fusarium solani*, *F. oxysporum* f. sp. *niveum*, *F. oxysporum* f. sp. *vasinfectum*, *G. saubinettii* (>100)<br>Positive control (MIC: µg/mL)<br>carbendazim: 12.5, 12.5, 6.25, 6.25, 25, 12.5, 25, 6.25, respectively<br>hymexazol: 12.5, 12.5, 12.5, 12.5, 50, 12.5, 2.5, 12.5, respectively	[32]
Fumiquinazoline F (**222**)	Antifungal activity (MIC: µg/mL)<br>*B. cinerea* (12.5), *A. solani* (25), *A. alternata* (12.5), *C. gloeosporioides* (12.5), *F. solani*(50), *F. oxysporum* f. sp. *niveum* (25), *F. oxysporum* f. sp. *vasinfectum* (50), *G. saubinettii* (12.5)<br>Positive control (MIC: µg/mL)<br>carbendazim: 12.5, 12.5, 6.25, 6.25, 25, 12.5, 25, 6.25, respectively<br>hymexazol: 12.5, 12.5, 12.5, 12.5, 50, 12.5, 2.5, 12.5, respectively	[32]
Fumiquinazoline G (**223**)	Antifungal activity (MIC: µg/mL)<br>*B. cinerea* (12.5), *A. solani* (25), *A. alternata* (12.5), *C. gloeosporioides* (12.5), *F. solani*(50), *F. oxysporum* f. sp. *niveum* (50), *F. oxysporum* f. sp. *vasinfectum* (50), *G. saubinettii* (12.5)<br>Positive control (MIC: µg/mL)<br>carbendazim: 12.5, 12.5, 6.25, 6.25, 25, 12.5, 25, 6.25, respectively<br>hymexazol: 12.5, 12.5, 12.5, 12.5, 50, 12.5, 2.5, 12.5, respectively	[32]
Fumiquinazoline D (**224**)	Antifungal activity (MIC: µg/mL)<br>*B. cinerea* (25), *A. solani* (25), *A. alternata* (25), *C. gloeosporioides* (25), *F. solani*(>100), *F. oxysporum* f. sp. *niveum* (50), *F. oxysporum* f. sp. *vasinfectum* (50), *G. saubinettii* (25)<br>Positive control (MIC: µg/mL)<br>carbendazim: 12.5, 12.5, 6.25, 6.25, 25, 12.5, 25, 6.25, respectively<br>hymexazol: 12.5, 12.5, 12.5, 12.5, 50, 12.5, 2.5, 12.5, respectively	[32]
Fumiquinazoline A (**225**)	Antifungal activity (MIC: µg/mL)<br>*B. cinerea* (12.5), *A. solani* (12.5), *A. alternata* (12.5), *C. gloeosporioides* (12.5), *F. solani*(50), *F. oxysporum* f. sp. *niveum* (25), *F. oxysporum* f. sp. *vasinfectum* (50), *G. saubinettii* (12.5)	[32]

	Positive control (MIC: μg/mL)<br>carbendazim: 12.5, 12.5, 6.25, 6.25, 25, 12.5, 25, 6.25, respectively<br>hymexazol: 12.5, 12.5, 12.5, 12.5, 50, 12.5, 2.5, 12.5, respectively	
3-hydroxyfumiquinazoline A (**226**)	Antifungal activity (MIC: μg/mL)<br>*B. cinerea* (12.5), *A. solani* (12.5),<br>*A. alternata* (12.5), *C. gloeosporioides* (12.5), *F. solani*(100), *F. oxysporum* f. sp. *niveum* (50),<br>*F. oxysporum* f. sp. *vasinfectum* (50), *G. saubinettii* (12.5)<br>Positive control (MIC: μg/mL)<br>carbendazim: 12.5, 12.5, 6.25, 6.25, 25, 12.5, 25, 6.25, respectively<br>hymexazol: 12.5, 12.5, 12.5, 12.5, 50, 12.5, 2.5, 12.5, respectively	[32]
6-methoxyspirotryprostatin B (**227**)	Antifungal activity (MIC: μg/mL)<br>*B. cinerea*, *A. solani*, *A. alternata*, *C. gloeosporioides*, *Fusarium solani*, *F. oxysporum* f. sp. *niveum*, *F. oxysporum* f. sp. *vasinfectum*, *G. saubinettii* (>100)<br>Positive control (MIC: μg/mL)<br>carbendazim: 12.5, 12.5, 6.25, 6.25, 25, 12.5, 25, 6.25, respectively<br>hymexazol: 12.5, 12.5, 12.5, 12.5, 50, 12.5, 2.5, 12.5, respectively	[32]
Spiro [5H,10H-dipyrrolo[1,2-α:1′,2′-d]pyrazine-2-(3H),2′-[2H]indole]-3′,5,10(1′H)-trione (**228**)	Antifungal activity (MIC: μg/mL)<br>*B. cinerea*, *A. solani*, *A. alternata*, *C. gloeosporioides*, *Fusarium solani*, *F. oxysporum* f. sp. *niveum*, *F. oxysporum* f. sp. *vasinfectum*, *G. saubinettii* (>100)<br>Positive control (MIC: μg/mL)<br>carbendazim: 12.5, 12.5, 6.25, 6.25, 25, 12.5, 25, 6.25, respectively<br>hymexazol: 12.5, 12.5, 12.5, 12.5, 50, 12.5, 2.5, 12.5, respectively	[32]
Pseurotin A (**229**)	Antifungal activity (MIC: μg/mL)<br>*B. cinerea*, *A. solani*, *A. alternata*, *C. gloeosporioides*, *Fusarium solani*, *F. oxysporum* f. sp. *niveum*, *F. oxysporum* f. sp. *vasinfectum*, *G. saubinettii* (>100)<br>Positive control (MIC: μg/mL)<br>carbendazim: 12.5, 12.5, 6.25, 6.25, 25, 12.5, 25, 6.25, respectively<br>hymexazol: 12.5, 12.5, 12.5, 12.5, 50, 12.5, 2.5, 12.5, respectively	[32]<br>[32]
Pseurotin A_1 (**230**)	Antifungal activity (MIC: μg/mL)<br>*B. cinerea*, *A. solani*, *A. alternata*, *C. gloeosporioides*, *Fusarium solani*, *F. oxysporum* f. sp. *niveum*, *F. oxysporum* f. sp. *vasinfectum*, *G. saubinettii* (>100)<br>Positive control (MIC: μg/mL)<br>carbendazim: 12.5, 12.5, 6.25, 6.25, 25, 12.5, 25, 6.25, respectively<br>hymexazol: 12.5, 12.5, 12.5, 12.5, 50, 12.5, 2.5, 12.5, respectively	[32]
Tryptoquivaline O (**231**)	Antifungal activity (MIC: μg/mL)<br>*B. cinerea* (25), *A. solani* (12.5), *A. alternata* (25), *C. gloeosporioides* (100), *F. solani*(>100),<br>*F. oxysporum* f. sp. *niveum* (>100), *F. oxysporum* f. sp. *vasinfectum* (>100), *G. saubinettii* (50)<br>Positive control (MIC: μg/mL)<br>carbendazim: 12.5, 12.5, 6.25, 6.25, 25, 12.5, 25, 6.25, respectively<br>hymexazol: 12.5, 12.5, 12.5, 12.5, 50, 12.5, 2.5, 12.5, respectively	[32]
Fumifaclavine B (**232**)	Antifungal activity (MIC: μg/mL)	[32]

	B. cinerea, *A. solani*, *A. alternata*, *C. gloeosporioides*, *Fusarium solani*, *F. oxysporum* f. sp. *niveum*, *F. oxysporum* f. sp. *vasinfectum*, *G. saubinettii* (>100)<br>Positive control (MIC: μg/mL)<br>carbendazim: 12.5, 12.5, 6.25, 6.25, 25, 12.5, 25, 6.25, respectively<br>hymexazol: 12.5, 12.5, 12.5, 12.5, 50, 12.5, 2.5, 12.5, respectively	
Bisdethiobis(methylthio)gliotoxin (**233**)	Antifungal activity (MIC: μg/mL)<br>*B. cinerea*, *A. solani*, *A. alternata*, *C. gloeosporioides*, *Fusarium solani*, *F. oxysporum* f. sp. *niveum*, *F. oxysporum* f. sp. *vasinfectum*, *G. saubinettii* (>100)<br>Positive control (MIC: μg/mL)<br>carbendazim: 12.5, 12.5, 6.25, 6.25, 25, 12.5, 25, 6.25, respectively<br>hymexazol: 12.5, 12.5, 12.5, 12.5, 50, 12.5, 2.5, 12.5, respectively	[32]
Cyclo-(Pro-Gly) (**234**)	Antifungal activity (MIC: μg/mL)<br>*B. cinerea*, *A. solani*, *A. alternata*, *C. gloeosporioides*, *Fusarium solani*, *F. oxysporum* f. sp. *niveum*, *F. oxysporum* f. sp. *vasinfectum*, *G. saubinettii* (>100)<br>Positive control (MIC: μg/mL)<br>carbendazim: 12.5, 12.5, 6.25, 6.25, 25, 12.5, 25, 6.25, respectively<br>hymexazol: 12.5, 12.5, 12.5, 12.5, 50, 12.5, 2.5, 12.5, respectively	[32]
Cyclo-(Pro-Ala) (**235**)	Antifungal activity (MIC: μg/mL)<br>*B. cinerea*, *A. solani*, *A. alternata*, *C. gloeosporioides*, *Fusarium solani*, *F. oxysporum* f. sp. *niveum*, *F. oxysporum* f. sp. *vasinfectum*, *G. saubinettii* (>100)<br>Positive control (MIC: μg/mL)<br>carbendazim: 12.5, 12.5, 6.25, 6.25, 25, 12.5, 25, 6.25, respectively<br>hymexazol: 12.5, 12.5, 12.5, 12.5, 50, 12.5, 2.5, 12.5, respectively	[32]
Cyclo-(D-Pro-L-Ala) (**236**)	Antifungal activity (MIC: μg/mL)<br>*B. cinerea*, *A. solani*, *A. alternata*, *C. gloeosporioides*, *Fusarium solani*, *F. oxysporum* f. sp. *niveum*, *F. oxysporum* f. sp. *vasinfectum*, *G. saubinettii* (>100)<br>Positive control (MIC: μg/mL)<br>carbendazim: 12.5, 12.5, 6.25, 6.25, 25, 12.5, 25, 6.25, respectively<br>hymexazol: 12.5, 12.5, 12.5, 12.5, 50, 12.5, 2.5, 12.5, respectively	[32]
Cyclo-(Pro-Ser) (**237**)	Antifungal activity (MIC: μg/mL)<br>*B. cinerea*, *A. solani*, *A. alternata*, *C. gloeosporioides*, *Fusarium solani*, *F. oxysporum* f. sp. *niveum*, *F. oxysporum* f. sp. *vasinfectum*, *G. saubinettii* (>100)<br>Positive control (MIC: μg/mL)<br>carbendazim: 12.5, 12.5, 6.25, 6.25, 25, 12.5, 25, 6.25, respectively<br>hymexazol: 12.5, 12.5, 12.5, 12.5, 50, 12.5, 2.5, 12.5, respectively	[32]
Cyclo-(Ser-trans-4-OH-Pro) (**238**)	Antifungal activity (MIC: μg/mL)<br>*B. cinerea*, *A. solani*, *A. alternata*, *C. gloeosporioides*, *Fusarium solani*, *F. oxysporum* f. sp. *niveum*, *F. oxysporum* f. sp. *vasinfectum*, *G. saubinettii* (>100)<br>Positive control (MIC: μg/mL)<br>carbendazim: 12.5, 12.5, 6.25, 6.25, 25, 12.5, 25, 6.25, respectively<br>hymexazol: 12.5, 12.5, 12.5, 12.5, 50, 12.5, 2.5, 12.5, respectively	[32]
Cyclo-(Leu-4-OH-Pro) (**239**)	Antifungal activity (MIC: μg/mL)	[32]

	B. cinerea, *A. solani*, *A. alternata*, *C. gloeosporioides*, *Fusarium solani*, *F. oxysporum* f. sp. *niveum*, *F. oxysporum* f. sp. *vasinfectum*, *G. saubinettii* (>100)<br>Positive control (MIC: μg/mL)<br>carbendazim: 12.5, 12.5, 6.25, 6.25, 25, 12.5, 25, 6.25, respectively<br>hymexazol: 12.5, 12.5, 12.5, 12.5, 50, 12.5, 2.5, 12.5, respectively	
Cyclo-(Ala-trans-4-OH-Pro) (**240**)	Antifungal activity (MIC: μg/mL)<br>*B. cinerea*, *A. solani*, *A. alternata*, *C. gloeosporioides*, *Fusarium solani*, *F. oxysporum* f. sp. *niveum*, *F. oxysporum* f. sp. *vasinfectum*, *G. saubinettii* (>100)<br>Positive control (MIC: μg/mL)<br>carbendazim: 12.5, 12.5, 6.25, 6.25, 25, 12.5, 25, 6.25, respectively<br>hymexazol: 12.5, 12.5, 12.5, 12.5, 50, 12.5, 2.5, 12.5, respectively	[32]
Cyclo-(cis-OH-D-Pro-L-Phe) (**241**)	Antifungal activity (MIC: μg/mL)<br>*B. cinerea*, *A. solani*, *A. alternata*, *C. gloeosporioides*, *Fusarium solani*, *F. oxysporum* f. sp. *niveum*, *F. oxysporum* f. sp. *vasinfectum*, *G. saubinettii* (>100)<br>Positive control (MIC: μg/mL)<br>carbendazim: 12.5, 12.5, 6.25, 6.25, 25, 12.5, 25, 6.25, respectively<br>hymexazol: 12.5, 12.5, 12.5, 12.5, 50, 12.5, 2.5, 12.5, respectively	[32]
Cyclo-(Gly-Phe) (**242**)	Antifungal activity (MIC: μg/mL)<br>*B. cinerea*, *A. solani*, *A. alternata*, *C. gloeosporioides*, *Fusarium solani*, *F. oxysporum* f. sp. *niveum*, *F. oxysporum* f. sp. *vasinfectum*, *G. saubinettii* (>100)<br>Positive control (MIC: μg/mL)<br>carbendazim: 12.5, 12.5, 6.25, 6.25, 25, 12.5, 25, 6.25, respectively<br>hymexazol: 12.5, 12.5, 12.5, 12.5, 50, 12.5, 2.5, 12.5, respectively	[32]
Cyclo-(Pro-*tans*-4-OH-Pro) (**243**)	Antifungal activity (MIC: μg/mL)<br>*B. cinerea*, *A. solani*, *A. alternata*, *C. gloeosporioides*, *Fusarium solani*, *F. oxysporum* f. sp. *niveum*, *F. oxysporum* f. sp. *vasinfectum*, *G. saubinettii* (>100)<br>Positive control (MIC: μg/mL)<br>carbendazim: 12.5, 12.5, 6.25, 6.25, 25, 12.5, 25, 6.25, respectively<br>hymexazol: 12.5, 12.5, 12.5, 12.5, 50, 12.5, 2.5, 12.5, respectively	[32]
Cyclo-(Gly-Ala) (**244**)	Antifungal activity (MIC: μg/mL)<br>*B. cinerea*, *A. solani*, *A. alternata*, *C. gloeosporioides*, *Fusarium solani*, *F. oxysporum* f. sp. *niveum*, *F. oxysporum* f. sp. *vasinfectum*, *G. saubinettii* (>100)<br>Positive control (MIC: μg/mL)<br>carbendazim: 12.5, 12.5, 6.25, 6.25, 25, 12.5, 25, 6.25, respectively<br>hymexazol: 12.5, 12.5, 12.5, 12.5, 50, 12.5, 2.5, 12.5, respectively	[32]
Uracil (**245**)	Antifungal activity (MIC: μg/mL)<br>*B. cinerea*, *A. solani*, *A. alternata*, *C. gloeosporioides*, *Fusarium solani*, *F. oxysporum* f. sp. *niveum*, *F. oxysporum* f. sp. *vasinfectum*, *G. saubinettii* (>100)<br>Positive control (MIC: μg/mL)<br>carbendazim: 12.5, 12.5, 6.25, 6.25, 25, 12.5, 25, 6.25, respectively<br>hymexazol: 12.5, 12.5, 12.5, 12.5, 50, 12.5, 2.5, 12.5, respectively	[32]

Table S3. Cytotoxic Activity of compounds (**165–174)** against Several Cells [59].

**Cell**	**Compound**										
	**165**	**166**	**167**	**168**	**169**	**170**	**171**	**172**	**173**	**174**	**Cont.**
A375 (IC_{50} μM)	>100	>100	34.4	47.4	48.5	71.2	18.0	72.9	41.7	42	18.1
AGS (IC_{50} μM)	>100	80.2	21.8	94.2	69.8	82.2	91.5	55.0	27.4	92.4	15.4
HCT-8 (IC_{50} μM)	>100	62.2	>100	7.5	4.4	4.2	1.0	0.3	4.7	0.6	22.0
HCT-8/T (IC_{50} μM)	>100	64.3	11.2	8.6	6.4	6.5	1.5	1.0	7.2	0.8	28.2
A549 (IC_{50} μM)	>100	42.9	27.6	6.4	4.0	3.3	1.0	0.6	2.0	0.6	12.1
MDA-MB-231(IC_{50} μM)	>100	62.8	44.6	3.4	6.3	5.5	7.5	1.0	3.7	1.0	6.3
SMMC-7721 (IC_{50} μM)	>100	87.4	29.3	>100	76.0	99.8	>100	68.5	50.8	>100	10.7
A2780 (IC_{50} μM)	>100	61.1	8.6	7.1	3.8	<1	<1	<1	<1	<1	8.5
